# Mesenchymal stem cell treatment improves outcome of COVID-19 patients via multiple immunomodulatory mechanisms

**DOI:** 10.1038/s41422-021-00573-y

**Published:** 2021-10-26

**Authors:** Rongjia Zhu, Tingdong Yan, Yingmei Feng, Yan Liu, Hongcui Cao, Gongxin Peng, Yanlei Yang, Zhen Xu, Jingqi Liu, Wei Hou, Xiaoyue Wang, Zhe Li, Luchan Deng, Shihua Wang, Jing Li, Qin Han, Hongling Li, Guangliang Shan, Yinghao Cao, Xingyan An, Jianshe Yan, Zhonghui Zhang, Huafei Li, Xuebin Qu, Jiaqi Zhu, Shumin Zhou, Jiao Wang, Fengchun Zhang, Jinming Gao, Ronghua Jin, Dayong Xu, Yan-Qing Ma, Tao Huang, Shuang Peng, Zhi Zheng, Ilia Stambler, Eric Gilson, Lee Wei Lim, Alexey Moskalev, Antonio Cano, Sasanka Chakrabarti, Brun Ulfhake, Huanxing Su, Haoying Xu, Sihuan Xu, Feng Wei, Holly M. Brown-Borg, Kyung-Jin Min, Georgina Ellison-Hughes, Calogero Caruso, Kunlin Jin, Robert Chunhua Zhao

**Affiliations:** 1 Institute of Basic Medical Sciences, Chinese Academy of Medical Sciences, School of Basic Medicine, Peking Union Medical College, Beijing, China; 2 School of Life Sciences, Shanghai University, Shanghai, China; 3 You’an Hospital, Capital Medical University, Beijing, China; 4 Puren Hospital Affiliated to Wuhan University of Science and Technology, Wuhan, Hubei China; 5 State Key Laboratory for the Diagnosis and Treatment of Infectious Diseases, the First Affiliated Hospital, College of Medicine, Zhejiang University, Hangzhou, Zhejiang China; 6 National Clinical Research Center for Infectious Diseases, Hangzhou, Zhejiang China; 7 Center for Bioinformatics, Institute of Basic Medical Sciences, Chinese Academy of Medical Sciences & School of Basic Medicine, Peking Union Medical College, Beijing, China; 8 Department of Medicine, Peking Union Medical College Hospital, Chinese Academy of Medical Sciences and Peking Union Medical College, Beijing, China; 9 Versiti Blood Research Institute, Milwaukee, WI USA; 10 Shanghai Institute of Nutrition and Health, Chinese Academy of Sciences, Shanghai, China; 11 Qingdao Walson Standard Biopharmaceutical Co, Ltd, Qingdao, Shangdong China; 12 International Society on Aging and Disease, Bryan, TX USA; 13 Department of Science, Technology and Society, Bar Ilan University, Ramat Gan, Israel; 14 Université Côte d’Azur, CNRS, Inserm, IRCAN, Faculty of Medicine, Nice, France; 15 Department of Medical Genetics, Centre Hospitalier Universitaire (CHU), Nice, France; 16 School of Biomedical Sciences, Li Ka Shing Faculty of Medicine, University of Hong Kong, Hong Kong, China; 17 Institute of Biology, Komi Science Center of Russian Academy of Sciences, Syktyvkar, Russia; 18 Russian Gerontological Research Clinical Center, Moscow, Russia; 19 Department of Pediatrics, Obstetrics and Gynecology, University of Valencia, Valencia, Spain; 20 Maharishi Markandeshwar Deemed University, Mullana-Ambala, India; 21 Karolinska University Hospital, Stockholm, Sweden; 22 Institute of Chinese Medical Science, University of Macau, Taipa, Macau, China; 23 State Key Laboratory of Advanced Materials for Smart Sensing, GRINM GROUP Co, Ltd, Beijing, China; 24 Department of Biomedical Sciences, University of North Dakota, School of Medicine & Health Sciences, Grand Forks, ND USA; 25 Department of Biological Sciences, Inha University, Incheon, Republic of Korea; 26 School of Basic and Medical Biosciences, Faculty of Life Sciences & Medicine, King’s College London, London, UK; 27 Department of Biomedicine, Neuroscience and Advanced Diagnostics, University of Palermo, Palermo, Italy; 28 University of North Texas Health Science Center, Bryan, TX USA

**Keywords:** Mechanisms of disease, Mesenchymal stem cells

## Abstract

The infusion of coronavirus disease 2019 (COVID-19) patients with mesenchymal stem cells (MSCs) potentially improves clinical symptoms, but the underlying mechanism remains unclear. We conducted a randomized, single-blind, placebo-controlled (29 patients/group) phase II clinical trial to validate previous findings and explore the potential mechanisms. Patients treated with umbilical cord-derived MSCs exhibited a shorter hospital stay (*P* = 0.0198) and less time required for symptoms remission (*P* = 0.0194) than those who received placebo. Based on chest images, both severe and critical patients treated with MSCs showed improvement by day 7 (*P* = 0.0099) and day 21 (*P* = 0.0084). MSC-treated patients had fewer adverse events. MSC infusion reduced the levels of C-reactive protein, proinflammatory cytokines, and neutrophil extracellular traps (NETs) and promoted the maintenance of SARS-CoV-2-specific antibodies. To explore how MSCs modulate the immune system, we employed single-cell RNA sequencing analysis on peripheral blood. Our analysis identified a novel subpopulation of VNN2^+^ hematopoietic stem/progenitor-like (HSPC-like) cells expressing CSF3R and PTPRE that were mobilized following MSC infusion. Genes encoding chemotaxis factors — CX3CR1 and L-selectin — were upregulated in various immune cells. MSC treatment also regulated B cell subsets and increased the expression of costimulatory CD28 in T cells in vivo and in vitro. In addition, an in vivo mouse study confirmed that MSCs suppressed NET release and reduced venous thrombosis by upregulating kindlin-3 signaling. Together, our results underscore the role of MSCs in improving COVID-19 patient outcomes via maintenance of immune homeostasis.

## Introduction

Coronavirus disease 2019 (COVID-19) is caused by severe acute respiratory syndrome coronavirus 2 (SARS-CoV-2).^1^ Although SARS-CoV-2 pathogenesis remains largely unexplored, disease severity is thought to arise from an overaggressive immune response compounded by inflammatory cell infiltration and increased production of inflammatory cytokines/chemokines. Indeed, recent studies have confirmed that patients with COVID-19 exhibit decreased numbers of peripheral blood lymphocytes and increased levels of serum proinflammatory cytokines.^2^ Currently, specific antiviral drugs for COVID-19 are still under development. Therefore, during the pandemic, there exists an urgent need for effective therapeutic strategies targeting the hyperactive inflammatory response of patients with COVID-19.

Although the overall mortality rate of COVID-19 differs among countries and is relatively low in some nations, each population has vulnerable groups for whom severe disease leads to acute respiratory distress syndrome (ARDS) and/or various cardiac complications, such as ventricular arrhythmia or acute coronary syndrome.^3^ These vulnerable groups usually comprise the aged population with pre-existing comorbidities, such as diabetes, cardiac dysfunction, and chronic kidney disease. The mortality rate in these patients is very high. Notably, two cellular aging hallmarks — immunosenescence and critical telomere shortening in peripheral blood mononuclear cells (PBMCs) — may increase the vulnerability of these patients.^4–7^ Thus, the major problem with treatment lies in the management of severe cases, especially those cases involving the elderly. The exaggerated immune response to the virus is largely responsible for severe cases of COVID-19, and this problem could be ameliorated by mesenchymal stem cell (MSC) therapy. New therapeutic strategies for COVID-19 are urgently needed to reduce the loss of lives during the spreading pandemic, because the vaccination of a significant portion of any population requires considerable time and effort. The targeting of the hyperactive systemic inflammatory response by MSC therapy, thus, opens up a new therapeutic avenue for COVID-19.

MSCs comprise a heterogeneous population that also show promise for tissue regeneration.^8^ MSCs were first discovered from observations of cultured human bone-marrow cell suspensions, which had lost hematopoietic potential in favor of the formation of proliferating adherent colonies of fibroblast-like cells with the potential to differentiate into adipocytes, chondrocytes and osteocytes, in vitro and in vivo.^9,10^ Although culture-expanded MSCs have been the focus of many studies, definitive MSC characterization and biology remain unclear.

The unique capacity of MSCs to regulate both immunity (in an autologous/allergenic manner) and tissue repair, makes them an attractive therapeutic cell type for acute/chronic and severe immune disorders. Interestingly, a recent study revealed that MSCs fail to express the receptor for angiotensin-converting enzyme 2 (ACE2) and, thus, should be insusceptible to SARS-CoV-2 infection.^11^ Therefore, we posit that MSCs could substantially improve the outcomes of COVID-19 patients by modulating the immune response, decreasing the extent of lung-tissue injury and facilitating its repair, and eventually, relieving acute pulmonary edema.

Neutrophil extracellular traps (NETs) are extracellular web-like structures composed primarily of chromatin fibers and microbicidal granule components.^12^ NET levels in plasma are significantly increased in patients with ARDS associated with COVID-19^13,14^ and have the potential to promote immunothrombosis.^15^ However, it remains unknown whether MSCs can suppress NET release in COVID-19 patients.

In this study, we systematically evaluated the efficacy of MSCs in the treatment of COVID-19 and assessed the mechanisms by which they regulated the immune molecular network and restored the immune system. We also investigated their role in promoting lung-tissue repair after severe pneumonia. Furthermore, we performed molecular analysis of PBMCs by using single-cell RNA sequencing (scRNA-seq) and related approaches.

## Results

### MSC treatment improves outcomes of COVID-19 patients, reduces NETs, and promotes the production of SARS-CoV-2-specific antibodies

**Fig. 1:**
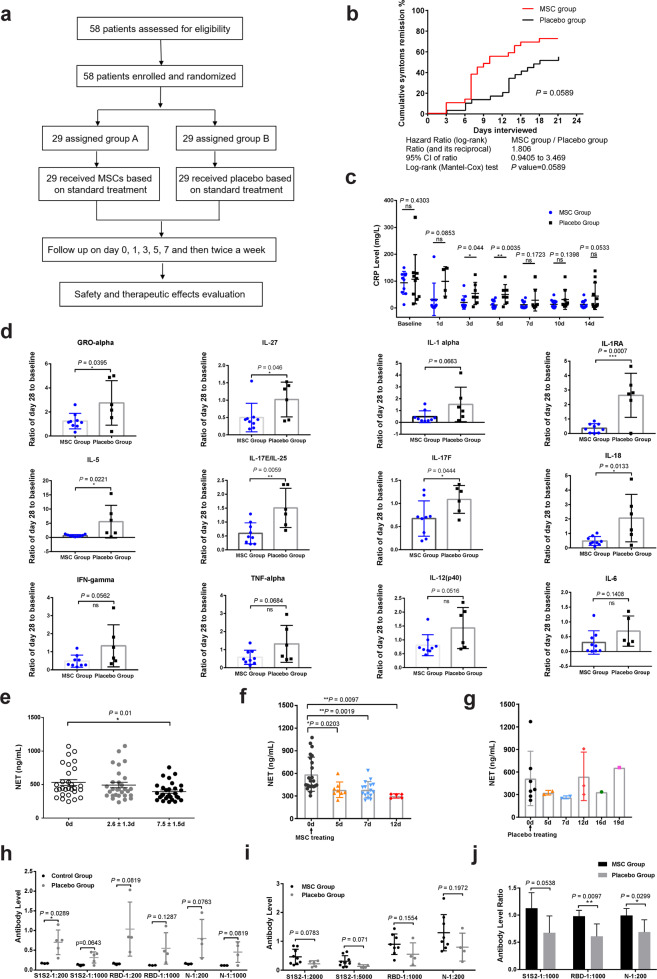
Clinical outcomes of COVID-19 patients with MSC transplantation. **a** Randomization and trial profile. **b** Cumulative remission rate of the two groups. **c** Plasma CRP levels were assessed for patients with severe/critical disease in the two groups. **d** Ratio of the mean value for each cytokine at day 28 to that of baseline (prior to treatment) after MSC or placebo infusion was calculated for the two groups. **e** Plasma NET-DNA levels for the MSC-treated patients at three time points (*n* = 29, *P* = 0.01, data at day 7.5 ± 1.5 compared with day 0). **f** Changes in the plasma NET-DNA levels in MSC-treated patients, showing the beneficial effects over time (*n* = 22, *P* = 0.0483, data at day 7 compared with that at day 0). **g** Change in plasma NET-DNA levels in placebo-treated patients over time (*n* = 7, *P* > 0.05). **h** Antibodies against SARS-CoV-2 spike S1 + S2 extracellular domain, RBD, and nucleocapsid/N detected in plasma of healthy subjects and placebo-treated patients over 28 days. **i** Detection of the three specific antibodies in plasma samples of both the MSC-treated and placebo groups on day 28 (*P* > 0.05). **j** Ratio of antibody level at day 28 to that of day 14 in the MSC-treated and placebo-treated groups. The data represent the means ± SD. The *P* values were determined using the unpaired Student’s *t-*test. *P < 0.05; **P < 0.01; ***P < 0.001.

Our previous clinical trial demonstrated the safety of transplantation of COVID-19 patients with ACE2^–^ MSCs, which substantially improved clinical outcomes.^11^ Thus, we conducted a randomized, single-blind, placebo-controlled phase II trial to further evaluate the safety and efficacy of transplantation. We enrolled 58 COVID-19 patients (22 men, 36 women) randomized into the MSC-treatment group or the placebo (normal saline) group (29 patients per group) with a 1:1 ratio (Fig. 1a). Among these 58 patients, 21 had severe disease, and 6 were critically ill. The baseline characteristics did not differ between the two groups of patients (Table 1); this was also true with respect to treatments received before and after MSC or placebo treatment (Table 2).

**Table 1:** Baseline characteristics of 58 patients with COVID-19.

Items	MSC group	Placebo group	*P* value	95% CI
Enrolled number				
	29	29	1.000	
Gender				
Men	12 (41.4)	10 (34.5)	0.7871	
Female	17 (58.6)	19 (65.5)	–	
Age				
Median	64 (54.5, 68)	66 (59.5, 69.5)	0.2221	−7.418 to 1.763
>50 (number, %)	24 (82.8)	28 (96.6)	0.194	
30–50 (number, %)	5 (17.2)	1 (3.4)	0.194	
COVID-19 type				
Common/mild	15 (51.7)	16 (55.2)	1.000	
Severe	11 (37.9)	10 (34.5)	1.000	
Critical	3 (10.3)	3 (10.3)	1.000	
Comorbidities				
Coronary heart disease	3 (10.3)	3 (10.3)	1.000	
Diabetes	4 (13.8)	4 (13.8)	1.000	
Cerebrovascular disease	3 (10.3)	2 (6.9)	1.000	
Hypertension	12 (41.4)	11 (37.9)	1.000	
Chronic respiratory disease	1 (3.4)	0 (0)	1.000	
History of liver and kidney disease	2 (6.9)	3 (10.3)	1.000	
Admission symptoms				
Cough	22 (75.9)	21 (72.4)	1.000	
Fever	16 (55.2)	20 (69.0)	0.417	
Anhelation	17 (58.6)	16 (55.2)	0.730	
Chest distress	11 (37.9)	14 (48.3)	0.596	
Fatigue	21 (72.4)	19 (65.5)	1.000	
Muscular soreness	9 (31.0)	5 (17.2)	0.358	
Poor appetite	3 (10.3)	5 (17.2)	0.787	
Diarrhea	3 (10.3)	3 (10.3)	1.000	
Dizziness	2 (6.9)	5 (17.2)	0.423	
Nausea and vomiting	3 (10.3)	3 (10.3)	1.000	
Time from symptom onset to starting study treatment				
Days	13 (9.5, 15.5)	11 (8, 14.5)	0.6908	−2.628 to 3.939
Admission laboratory data				
Total bilirubin (μmol/L)	11.1 (9.16, 15.2)	10.9 (9.05, 13.8)	0.6355	−3.722 to 2.292
C-reative protein (mg/L)	51.4 (18.3, 100.6)	55.2 (32.0, 110.2)	0.2648	−14.14 to 50.33
Procalcitonin (ng/mL)	0.10 (0.04, 0.14)	0.09 (0.04, 0.17)	0.2887	−0.1216 to 0.4002
WBC (/uL)	6.31 (4.20, 7.37)	6.75 (4.92, 8.64)	0.0781	−4.211 to 0.2313
Neutrophils (/uL)	5.66 (3.40, 7.48)	4.34 (2.91, 5.95)	0.0938	−3.142 to 0.2534
Lymphocyte (/uL)	0.64 (0.42, 1.12)	0.93 (0.54, 1.24)	0.1827	−0.08833 to 0.4521
Monocytes (/uL)	0.25 (0.19, 0.48)	0.30 (0.20, 0.44)	0.5397	−0.2566 to 0.4848
Hemoglobin (g/L)	130 (114, 145)	126 (119, 136)	0.8587	−8.995 to 7.521
Platelet (/uL)	162 (143–238)	208 (158, 254)	0.4755	−26.03 to 55.1
Alanine aminotransferase (U/L)	40.0 (29.5–63.4)	32.5 (23.2, 47.8)	0.8469	−26.15 to 21.54
Aspartate aminotransferase (U/L)	31.9 (27.3–47.5)	33.4 (23.5, 47.3)	0.7854	−20.64 to 15.69
Creatinine (mg/dL)	62.9 (47.0–80.5)	61.8 (53.1, 81.4)	0.1683	−118.6 to 21.23
Serum potassium (mmol/L)	3.73 (3.46–3.88)	3.79 (3.55, 4.13)	0.2792	−0.1147 to 0.3897
Serum sodium (mmol/L)	139 (137–141)	139 (135–141)	0.8151	−2.961 to 2.339
Activated partial prothrombin time (s)	27.5 (25.0–31.7)	28.9 (26.2–32.2)	0.5698	−1.655 to 2.975
Fibrinogen (g/s)	4.60 (3.42–4.98)	4.59 (3.94–5.17)	0.522	−0.4026 to 0.7837

Data are median (IQR) or *n* (%).

**Table 2:** Treatments received before and after enrollment.

Items	Baseline			Treatment		
	MSC	Placebo	*P* value	MSC	Placebo	*P* value
Concomitant treatment						
Oxygen therapy	27 (93.1)	24 (88.9)	0.423	26 (89.7)	27 (93.1)	1.000
Non-invasive mechanical	3 (10.3)	2 (6.9)	1.000	3 (10.3)	3 (10.3)	1.000
Invasive mechanical	0	0	–	0	2 (6.9)	0.491
Corticosteroids						
No. of patients	20 (70.0)	19 (65.5)	1.000	16 (55.2)	17 (58.6)	1.000
Median days	4 (3, 6)	4 (2, 7)	0.7525	4 (1, 9)	7 (5, 14)	0.2294
Doses (mg/d)	40 (40, 73.3)	40 (40, 80)	0.6547	24.4 (3, 41.7)	28.6 (13,3, 46.4)	0.7685
Antibiotic						
No. of patients	18 (62.1)	19 (65.5)	1.000	16 (55.2)	18 (62.1)	0.790
Moxifloxacin	12 (41.4)	18 (62.1)	0.189	8 (27.6)	16 (55.2)	0.061
Median days	4 (3.25, 6.75)	3 (1.25, 4.75)	0.1287	7 (0.5, 8.75)	8 (2.25, 10)	0.4577
Piperacillin tazobactam	10 (34.5)	8 (27.6)	0.777	8 (27.6)	5 (17.2)	0.530
Median days	6.5 (5, 9.75)	2.5 (1.25, 16.25)	0.8948	5 (0, 10.75)	3.5 (0, 7.75)	0.5512
Levofloxacin	4 (13.8)	3 (10.3)	1.000	3 (10.3)	2 (6.9)	1.000
Median days	3 (1, 5)	3 (0, 11.5)	0.833	3 (0, 6)	0 (0, 7.5)	–
Anti-virus therapy						
No. of patients	13 (44.8)	17 (58.6)	0.431	12 (41.4)	15 (51.7)	0.599
α-Interferon	5 (17.2)	9 (31.0)	0.358	5 (17.2)	9 (31.0)	0.358
Median days	5 (3, 10)	5 (3, 7.25)	0.5285	13 (10.5, 16.5)	10.5 (5.5, 15)	0.2938
Ribavirin	11 (37.9)	12 (41.4)	1.000	9 (31.0)	13 (44.8)	0.417
Median days	4.5 (1.5, 5.75)	2 (2, 4.5)	0.3917	5 (0.25, 7.25)	5 (5, 12)	0.0725
Ganciclovir	9 (31.0)	7 (24.1)	0.770	1 (3.4)	1 (3.4)	1.000
Median days	4 (3, 7)	2 (1, 15.5)	0.899	–	–	–

Data are median (IQR) or *n* (%).

For the primary endpoint, the median time of hospital stay for patients in the MSC group was shorter than that of the placebo group (11 days (interquartile range, 8–14) vs 15 days (interquartile range, 11–19); log-rank test *P* = 0.1380; hazard ratio, 1.693; 95% confidence interval, 0.8227–3.484) (Supplementary information, Fig. S1a), as confirmed by a *t*-test (*P* = 0.0198) (Table 3). Additionally, the median time of symptoms remission in the MSC group was also shorter than that of the placebo group (7 days (interquartile range, 7–12) vs 13 days (interquartile range, 8–16), *P* = 0.0194 by a *t*-test). MSC-treated patients achieved a better outcome of symptoms by day 7, 14 and 21 (*P* = 0.031, *P* = 0.0466 and *P* = 0.0187 by *χ*^2^ test) than placebo-treated patients (Table 3). The cumulative symptom remission rate was higher in the MSC-treated group than in the placebo group (log-rank test *P* = 0.0589; hazard ratio, 1.806; 95% confidence interval, 0.9405–3.469) (Fig. 1b). Notably, severe or critical patients achieved better symptoms outcome in the MSC-treated group than those in the placebo group by day 14 (*P* = 0.0405) and day 21 (*P* = 0.0157 by the *χ*^2^ test) (Supplementary information, Table S3). Moreover, follow-up computed tomography of the chest revealed that the diffuse density of both lungs of patients with severe or critical COVID-19 was significantly improved in the MSC group compared to the placebo group by day 7 (*P* = 0.0099) and day 21 (*P* = 0.0084; χ^2^ test) (Table 3). These results suggested that MSC can improve the symptoms of severe or critical patients significantly.

**Table 3:** Outcomes of treatment for the MSC-treated and placebo-treated patients.

Items	MSC group	Placebo group	*P* value^a^
*Clinical improvement rates*			
Day 7			0.031
Symptom remission^b^	11 (37.9)	4 (13.8)	
Improvement	17 (58.6)	19 (65.5)	
No improvement	1 (3.4)	6 (20.7)	
Day 14			0.0466
Symptom remission	19 (65.5)	12 (41.4)	
Improvement	9 (31.0)	10 (34.5)	
No improvement	1 (3.4)	7 (24.1)	
Day 21			0.0187
Symptom remission	21 (72.4)	16 (55.2)	
Improvement	8 (27.6)	6 (20.7)	
No improvement	0	7 (24.1)	
*Chest image results based on CT and X-ray*			
Patients with common/mild COVID-19			
Day 7			0.5756
Improvement	6 (20.7)	7 (24.1)	
Progression-free	8 (27.6)	9 (31.0)	
Progression	1 (3.4)	0	
Day 14			0.3171
Improvement	6 (20.7)	7 (24.1)	
Progression-free	7 (24.1)	9 (31.0)	
Progression	2 (6.9)	0	
Day 21			0.5436
Improvement	7 (24.1)	7 (24.1)	
Progression-free	7 (24.1)	9 (31.0)	
Progression	1 (3.4)	0	
Patients with severe/critical COVID-19			
Day 7			0.0099
Improvement	10 (34.5)	2 (6.7)	
Progression-free	4 (13.8)	9 (31.0)	
Progression	0	2 (6.7)	
Day 14			0.0754
Improvement	9 (31.0)	3 (10.3)	
Progression-free	4 (13.8)	6 (20.7)	
Progression	1 (3.4)	4 (13.8)	
Day 21			0.0084
Improvement	11 (37.9)	3 (10.3)	
Progression-free	3 (10.3)	6 (20.7)	
Progression	0	4 (13.8)	
Median time required for symptoms remission^c^			
	7 (7, 12)	13 (8, 16)	0.0194^d^
Median time of hospital stay^c^			
	11 (8, 14)	15 (11, 19)	0.0198^d^

Data are median (IQR) or *n* (%).

^a^*χ*^2^ test was used.

^b^This assessment includes patients whose symptoms have disappeared and patients discharged from hospital.

^c^Day 21 assessment after treatment.

^d^*t*-test was used.

For the secondary endpoint, we assessed the levels of serum C-reactive protein (CRP) in the two groups to determine whether the infusion with MSCs could modulate the immune system. The changes in CRP levels of the patients in the two groups were consistent with their treatment outcomes (Supplementary information, Fig. S2a–d). Notably, CRP levels were significantly decreased in patients with severe disease in the MSC group compared to patients in the placebo group, especially at day 3 (20.27 ± 7.604 mg/L vs 54.21 ± 15.53 mg/L, *P* = 0.044) and day 5 (10.82 ± 3.982 mg/L vs 50.16 ± 13.87 mg/L, *P* = 0.0035) (Fig. 1c). At day 28, the levels of plasma proinflammatory cytokines — IL-1RA, IL-18, IL-27, IL-17E/IL-25, IL-17F, GRO-alpha (CXCL-1), and IL-5 — were substantially lower in the MSC-treated patients than in the placebo group (*P* < 0.05) (Fig. 1d). The 28-day mortality rate was 0.0% for the MSC group, while it was 6.9% for the placebo group (Table 4).

**Table 4:** Adverse events recorded for the MSC-treated and placebo-treated patients.

Items	MSC group	Placebo group
Adverse events		
Number of patients	3 (10.3)	13 (44.8)
Disturbance of consciousness	0	2 (6.9)
Urinary tract infection	0	1 (3.4)
Headache	0	1 (3.4)
Palpitations	1 (3.4%)	3 (10.3)
Fever	0	3 (10.3)
Diarrhea/bloating	0	2 (6.9)
Inappetence	0	1 (3.4)
Increased blood pressure	1 (3.4)	2 (6.9)
Body pain	1 (3.4)	3 (10.3)
Lab examinations within 3 days		
Increased alanine aminotransferase	12 (41.4)	11 (37.9)
Hyperbilirubinemia	2 (6.9)	4 (13.8)
Increased creatinine	3 (10.3)	2 (6.9)
28-day mortality		
	0	2 (6.9)

Data are *n* (%).

Safety effects were assessed by supervising vital signs before and 24 h after treatment with MSCs or placebo. Temperature, pulse, breathing rate, and systolic and diastolic pressure were similar between the two groups (Table 5). More serious adverse events were recorded for the placebo group than for the MSC group; however, the difference was not statistically significant (Table 4).

**Table 5:** Assessment of vital signs of the MSC-treated and placebo-treated patients.

Items	Baseline			Treatment		
	MSC	Placebo	*P* value	MSC	Placebo	*P* value
Temperature (°C)	36.7 (36.5, 38.0)	36.6 (36.4, 36.8)	0.2625	36.5 (36.3, 36.6)	36.6 (36.4, 36.8)	0.0137
Pulse (times/min)	78 (75.0, 86)	80 (77, 90)	0.4701	77 (71, 80)	78 (75, 85)	0.3846
Breath (times/min)	20 (18, 21)	20 (19, 20)	0.9326	20 (18, 22)	20 (18, 20)	0.5586
Systolic pressure (mmHg)	130 (118, 136)	128 (119, 137)	0.7764	130 (121, 135)	130 (122, 135)	0.9433
Diastolic pressure (mmHg)	79 (75, 81)	74 (70, 80)	0.1491	78 (75, 80)	75 (70, 78)	0.0407

Data are median (IQR).

NETs are indicative of pathogenic immunothrombosis in COVID-19 patients.^16^ Circulating markers of NET formation in COVID-19 patients, such as cell-free DNA (NET-DNA) and citrullinated histone H3 (CitH3), are associated with clinical outcome.^17^ Therefore, we compared the levels of plasma NET-DNA before and after MSC treatment using the Sytox Green assay.^18,19^ Plasma NET-DNA was reduced 7.5 days after MSC treatment (395.91 ± 24.93 ng/mL vs 531.89 ± 42.83 ng/mL, *P* = 0.01) (Fig. 1e); the baseline levels of NET-DNA were comparable in the MSC and placebo groups (Supplementary information, Fig. S1b). We also performed ELISA to measure the NET complex of CitH3-DNA and observed similar results (Supplementary information, Fig. S1c). Further, we analyzed 22 of the 29 MSC-treated patients by collecting daily blood samples and found that their plasma NET-DNA levels steadily decreased over time (day 5 vs day 0, *P* = 0.0203; day 7 vs day 0, *P* = 0.0019; day 12 vs day 0, *P* = 0.0097) (Fig. 1f; Supplementary information, Fig. S1d). Negligible effect was observed in the placebo group (Fig. 1g; Supplementary information, Fig. S1e). These results suggest that MSC treatment can efficiently reduce the levels of plasma NETs in COVID-19 patients.

Human plasma antibodies that are specific for SARS-CoV-2 spike S1 + S2 extracellular domain, spike receptor-binding domain (RBD), and nucleocapsid/N were also monitored on days 14 and 28 after MSC treatment. On day 28, the levels of plasma antibodies against SARS-CoV-2 were moderately higher in the placebo group than in healthy control subjects (no COVID-19 diagnosis) (Fig. 1h). Importantly, the plasma levels of SARS-CoV-2 antibodies in MSC-treated patients were noticeably higher than those in the placebo group on day 28 (Fig. 1i). Moreover, the ratio of antibody levels between day 28 and day 14 in the MSC-treated group was ~1.0, which was higher than the ratio of ~0.5 in the placebo group (Fig. 1j). These results suggested that MSC treatment not only improved the clinical outcomes of COVID-19 patients but also reduced the levels of CRP, proinflammatory cytokines, and NETs, as well as promoted the production of SARS-CoV-2-specific antibodies and maintained their levels for a longer period compared with placebo treatment.

### High-throughput sequencing of peripheral blood cells from COVID-19 patients who received MSC infusion

**Fig. 2:**
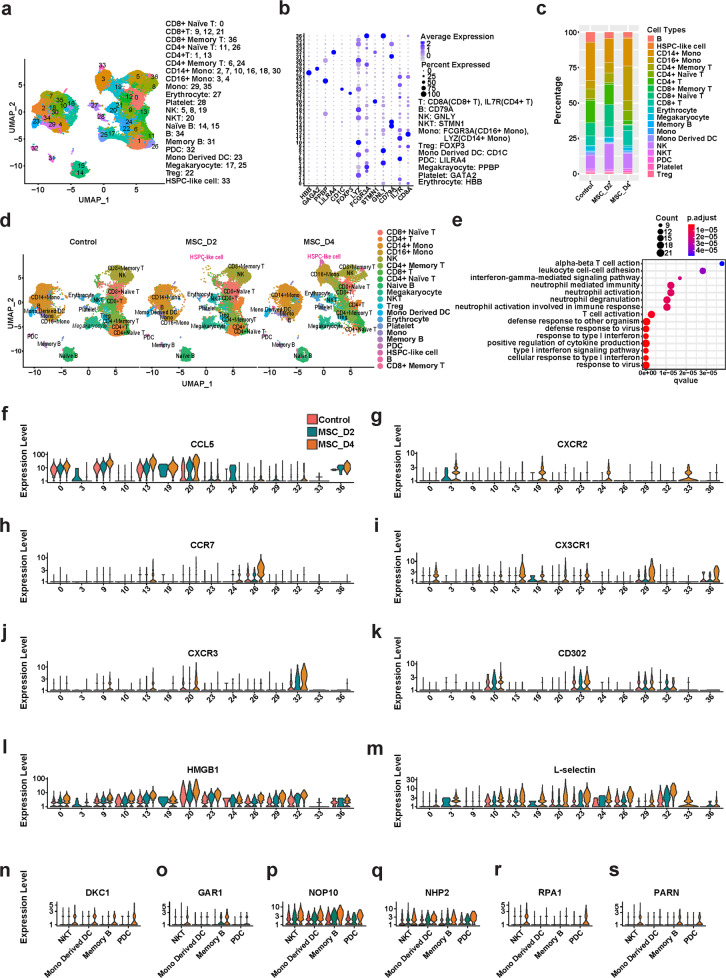
Expression of chemotaxis-related genes and telomerase-related genes in PBMCs of COVID-19 patients treated with MSCs by high-throughput sequencing. **a** UMAP presentation of major cell types and associated clusters among PBMCs of COVID-19 patients (*n* = 7). **b** Heatmap showing expression of hallmark genes stratified by cell clusters. The markers and their corresponding cell clusters are listed on the right. **c** Proportion of the major immune cell types among PBMCs from MSC-treated COVID-19 patients (MSC-D2 and MSC-D4) and MSC-untreated controls. **d** UMAP presentation of major cell types and associated clusters among PBMCs from MSC-treated COVID-19 patients (MSC-D2 and MSC-D4) and MSC-untreated controls. The HSPC-like cell cluster is highlighted in pink. **e** GO analysis of the top 171 most highly expressed genes in the bone marrow-derived cluster. Bubble chart showing the top 15 GO biological process terms. **f–m** DEG levels of CCL5, CXCR2, CCR7, CX3CR1, CXCR3, CD302, HMGB1, and L-selectin detected by scRNA-seq in different subpopulations of PBMCs from MSC-treated COVID-19 patients (MSC-D2 and MSC-D4) and MSC-untreated controls. **n–s** Differential gene expression levels of DKC1, GAR1, NOP10, NHP2, RPA1, and PARN in different leukocyte subpopulations of PBMCs from MSC-treated COVID-19 patients (MSC-D2 and MSC-D4) and MSC-untreated control samples. UMAP, Uniform Manifold Approximation and Projection; control, MSC-untreated controls.

To gain further insights into the mechanism by which MSC treatment modulates the immune response in COVID-19 patients, we analyzed 7 samples for scRNA-seq to characterize PBMCs: 6 samples from two COVID-19 patients on days 0, 2 and 4 after they were treated with MSCs, and 1 sample for one placebo-treated patient on day 0. The two day 0 samples from the two patients before MSC-treatment and one sample from the placebo-treated patient were considered as three MSC-untreated control samples. A total of 37 clusters (clusters 0–36) were identified (Fig. 2a), and the markers expressed in each of the clusters are shown in the heatmap (Supplementary information, Fig. S3a). Based on the expressions of CD8A, IL7R, CD79A, GNLY, STMN1, FCGR3A, LYZ, FOXP3, CD1C, LILRA4, PPBP, GATA2, and HBB (Fig. 2b; Supplementary information, Fig. S3b), the 37 clusters represented the following 21 major cell-type groups after annotation: CD8^+^ naïve T cells, CD8^+^ T cells, CD8^+^ memory T cells, CD4^+^ naïve T cells, CD4^+^ T cells, CD4^+^ memory T cells, CD14^+^ monocytes, CD16^+^ monocytes, monocytes, erythrocytes, platelets, natural killer (NK) cells, NK T cells, naïve B cells, B cells, memory B cells, plasmacytoid dendritic cells (pDCs), monocyte-derived DCs, megakaryocytes, regulatory T cells (Tregs), and HSPC-like cells. The PBMCs from MSC-treated COVID-19 patients contained higher proportions of CD16^+^ monocytes and lower proportions of CD4^+^ T and B cells than the placebo group (Fig. 2c). In contrast, the relative abundances of CD14^+^ monocytes decreased after both 2 days and 4 days of MSC treatment (Fig. 2c; Supplementary information, Fig. S3c, d). Interestingly, a recent study showed a significant increase of CD14^+^ monocytes and B cells in COVID-19 patients, which was shown to be associated with disease severity.^20^ Thus, our finding suggests that MSC treatment improved the outcomes of COVID-19 patients by modulating the immune composition of the peripheral blood of COVID-19 patients.

The scRNA-seq results revealed that subpopulation 33 increased continuously in the PBMC samples from patients after MSC treatment for 2 or 4 days (Fig. 2d). To explore the relevance of subpopulation 33, we assessed the enrichment of 171 highly expressed genes based on Gene Ontology (GO) analysis (Fig. 2e). These genes were found to be mainly involved in T cell activation, neutrophil-mediated immunity, upregulation of cytokine production, type I interferon (IFN-I) signaling, and response to virus challenge, all of which strongly suggested that this bone marrow -derived cell subpopulation may play vital roles in the activation and maintenance of the immune response to SARS-CoV-2 infection.

Interesting, this subpopulation included 171 highly expressed candidate genes, of which 13 (CSF3R, CXCR2, RICTOR, STAT3, VNN2, PTPRE, HIF1A, PTEN, TGFBR2, JUNB, IL17RA, TNFSF10, and FOS) were mainly associated with angiopoiesis, hematopoietic stem cell (HSC) mobilization, and fetal extramedullary hematopoiesis. Among the 13 functional genes, VNN2 is an important surface marker of human embryonic hematopoietic stem cells. It can be also used to isolate human embryonic hematopoietic stem cells, which has been confirmed by classic bone marrow transplantation experiments.^21^ These data indicated that a newly identified VNN2^+^ hematopoietic stem/progenitor like cell (HSPC-like cell) group was mobilized following MSC infusion. This suggested extramedullary hematopoiesis which may provide possible conditions for the subsequent activation of the immune function in the peripheral blood of COVID-19 patients.

Additionally, principal component analysis of SNP showed that the newly formed HSPC-like cells, CD4^+^ T and B cells, from the same sample of a single patient, were aggregated (Supplementary information, Fig. S3e), suggesting that the analyzed HSPC-like cells came from the same patient rather than the infused MSCs.

### MSCs promote the transcription of chemotaxis-related and telomerase-related genes in PBMCs of COVID-19 patients

The scRNA-seq analysis revealed that, compared with the placebo group, in the MSC group, elevated expressions of the 8 chemotaxis-related genes (*CCL5, CXCR2, CCR7, CX3CR1, CXCR3, CD302, HMGB1*, and *L-selectin*) were observed both after 2 and 4 days. These genes were upregulated in 14 clusters of immune cells, including monocytes, NK cells, DCs, and T cells. Specifically, CCL5 transcription was upregulated in CD4^+^ memory T cells (cluster 24), CD8^+^ memory T cells (cluster 36), CD16^+^ monocytes (cluster 3), and NK cells (cluster 19) (Fig. 2f). On day 4 of MSC treatment, high levels of CXCR2 were observed in CD4^+^ memory T cells (cluster 24), CD16^+^ monocytes (cluster 3), and NK cells (cluster 19) (Fig. 2g). The levels of the following factors were also increased on day 4: CCR7 in CD4^+^ naïve T cells (cluster 26) (Fig. 2h); CX3CR1 in CD8^+^ naïve T cells (cluster 0), CD8 T cells (cluster 9), CD8 memory T cells (cluster 36), CD14^+^ monocytes (cluster 10), monocytes (cluster 29), NK cells (cluster 19), NKT cells (cluster 20), and DCs (cluster 23) (Fig. 2i); and CXCR3 in pDCs (cluster 32) (Fig. 2j). In addition to these chemokines and chemokine receptors, the transcription of three other important chemotaxis factors was upregulated: CD302 in pDCs (cluster 32) (Fig. 2k), HMGB1 in CD4^+^ T cells (cluster 13) and NK cells (cluster 19) (Fig. 2l), and L-selectin in CD16^+^ monocytes (cluster 3), monocytes (cluster 29), NK cells (cluster 19), and pDCs (cluster 32) (Fig. 2m).

We next investigated the expression of *hTERT* and several other genes involved in telomerase assembly and maturation.^22,23^ Owing to the small number of cells available, *hTERT* expression was below the detection limit. Compared with the placebo-treated patients, 2- and 4-day MSC treatment significantly increased the expression levels of *DKC1, GAR1, NOP10, NHP2, RPA1*, and *PARN* in memory B cell clusters (Fig. 2n–s). After 4 days of MSC treatment, the following were upregulated: *DKC1, NOP10*, and *NHP2* in monocyte-derived DC clusters (Fig. 2n, p, q) and *DKC1, GAR1, NOP10, NHP2, RPA1* and *PARN* in NKT cell clusters (Fig. 2n–s).

Together, these results suggested that MSCs, upon infusion into patients, can modulate the development, activation, and chemotaxis of DCs, T cells, and B cells by upregulating specific genes.

### MSC treatment promotes immune regulatory function

**Fig. 3:**
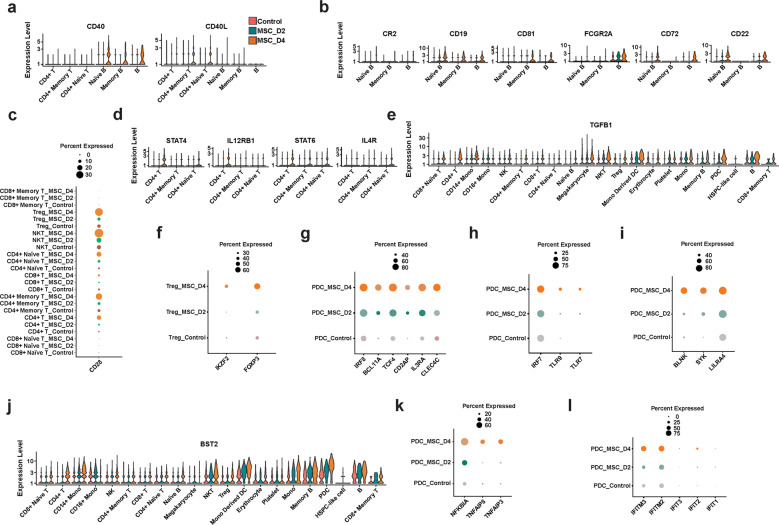
MSC treatment promotes immune regulatory functions. **a–d** Expression of markers in various cell types in peripheral blood. **a** CD40 and CD40L in B cells and CD4^+^ T cells; **b** CR2, CD19, CD81, FCGR2A, CD72, and CD22 in B cells; **c** CD28 in T cells; **d** STAT4, IL12RB1, STAT6, and IL4R in CD4^+^ T cells; **e** TGF-β1 in various immune cells. **f** Expression of the Treg-specific genes FOXP3 and IKZF2 in samples MSC-D2 and MSC-D4. **g** Expression of the pDC-specific genes CLEC4C, IL-3Rα, and CD2AP and regulatory genes TCF4, BCL11A, and IRF8. **h** Expression of TLR7 and TLR9 and of the important IFN-I regulator IRF7. **i** Expression of the pDC regulator receptor LILRA4 and its downstream signaling genes BLNK and SYK. **j** Expression of BST2 in various immune cells in peripheral blood. **k** Expression of the NFκB negative regulators TNFAIP3, TNFAIP8, and NFKBIA. **l** Expression of the IFN downstream genes IFIT1, IFIT2, IFIT3, IFITM2, and IFITM3; control, MSC-untreated controls.

As plasma antibodies for SARS-CoV-2 spike S1 + S2 extracellular domain, RBD, and nucleocapsid/N were found to be elevated and maintained for a longer time in MSC-treated patients than in placebo-treated patients (Fig. 1h–j), we studied B cell activation after MSC treatment. The proportions of naïve and intermediate-stage B cells were significantly decreased, whereas the proportion of memory B cells was not significantly altered (Fig. 2d). B cell activation requires secondary signals via the engagement of costimulatory molecules such as CD40. The phenotypic analysis revealed that MSC treatment for 4 days respectively upregulated the expression of CD40 in naïve and intermediate-stage B cells and CD40L in CD4^+^ naïve T cells (Fig. 3a), indicating that B cells were more likely to undergo activation. The B cell costimulatory complex comprises CR2, CD19, and CD81, and this complex significantly lowers the antigen-binding threshold for the activated B cells. The remarkable upregulation of CD19 and CD81 after 4 days of MSC treatment (Fig. 3b) suggested that B cells were more responsive to SARS-CoV-2 infection. Interestingly, coinhibitory receptors, FCGR2A, CD72 and CD22 — which downregulate B-cell receptor (BCR) signaling and function by acting as a molecular switch — were also upregulated in B cells after MSC treatment (Fig. 3b). These results indicate that MSCs are proficient at modulating B cell activation at a reasonable level by upregulating both costimulatory and coinhibitory receptors in patient B cells, the results of which may not be realized by other immunomodulatory therapy. CD28 is critical and indispensable for multiple functions of T cells, including T cell activation and survival of diverse T cells. Moreover, loss of CD28 is associated with various immune disorders.^24–27^ We observed that CD28 expressed on CD4^+^ T cells, naïve T cells, NKT cells, CD4^+^ memory T cells, and Treg cells were remarkably enhanced after MSC infusion on days 2 and 4, suggesting that MSCs can promote T cell activation (Fig. 3c). Activation of B cells also requires the assistance of T helper (Th) cells. IL12/IL12R signaling activates STAT4 to promote the production of Th1 cells from CD4^+^ T cells, whereas IL4/IL4R signaling activates STAT6 to promote the differentiation of CD4^+^ T cells into Th2 cells. MSC treatment upregulated the expression of IL12R, STAT4, and STAT6 in CD4^+^ T cells and CD4^+^ naïve T cells (Fig. 3d). Moreover, STAT6 expression was also elevated in CD4^+^ memory T cells (Fig. 3d), suggesting that MSCs can promote the differentiation of CD4^+^ T cells into Th cells to promote B-cell activation, leading to the production of SARS-CoV-2-specific antibodies.

MSC treatment also had immunosuppressive effects on other functions of PBMCs. The gene encoding the immune suppressor TGF-β1 was upregulated in CD4^+^ T cells, CD8^+^ T cells, monocytes, NKT cells, B cells, DCs, and pDCs (Fig. 3e). Tregs are an important subgroup of lymphocytes that suppress the immune response. On day 4 after MSC treatment, the *FOXP3* gene was dramatically upregulated in Tregs. *IKZF2* (*Helios)*, which enhances Treg function in cooperation with *FoxP3*, was also upregulated (Fig. 3f).

pDC cells produce large amounts of IFNs in response to viral infection.^28^ pDC-specific markers CLEC4C (also known as BDCA-2), IL-3Rα (CD123), and CD2AP were highly expressed after MSC treatment. The committed differentiation regulatory gene *TCF4* (*E2-2)* and its target genes (*BCL11A and IRF8)* were also upregulated on day 4 after MSC treatment (Fig. 3g), as were the important IFN-I regulator, IRF7, and the endosomal pattern recognition receptors, TLR7 and TLR9, which are activated by viral nucleic acids (Fig. 3h). The vital inhibitory receptor, LILRA4 (ILT7), of pDCs can be activated by BST2 to inhibit IFN and proinflammatory cytokine production by pDCs.^29^ Interestingly, MSC treatment greatly increased the cellular levels of both LILRA4 and BST2 (Fig. 3i, j). The pDC regulatory receptors — ILT7 and BDCA-2 — can signal through the immunoreceptor tyrosine-based activation motif (ITAM) pathway and involve spleen tyrosine kinase (SYK) and adaptor protein B-cell linker (BLNK). The expressions of both SYK and BLNK were increased following MSC treatment (Fig. 3i). Moreover, negative regulators of NF-κB, TNFAIP3, TNFAIP8 and NFKBIA were upregulated in pDCs after MSC treatment (Fig. 3k), whereas the IFN downstream genes, *IFIT1, IFIT2, IFIT3, IFITM2* and *IFITM3*, were slightly upregulated (Fig. 3l).

Together, these results suggest that MSCs can potently alter PBMC functions, as evidenced by the increased level of TGF-β1 in various immune cells, the upregulation of *FOXP3* and *IKZF2* in Tregs, and suppression of IFN-I production in pDCs.

To delineate the responsive pathways associated with MSC treatment, we performed differential co-expression analyses. For each cell type, we enriched the top 500 most highly expressed genes to yield 332 genes that interact with SARS-CoV-2 using the hypergeometric test. It was found that the network on day 2 was significantly rewired, with many new activated protein–protein interactions. On day 4, however, the network had largely been restored to that on day 0 (Supplementary information, Fig. S4). These results suggest that dramatic changes occur during the early stage of MSC treatment, demonstrating that MSCs are potent modulators of the immune response to SARS-CoV-2.

### MSCs regulate the spectrum of T-cell subtypes and promote co-stimulator CD28 expression partially via MAPK-ERK/JNK signaling

**Fig. 4:**
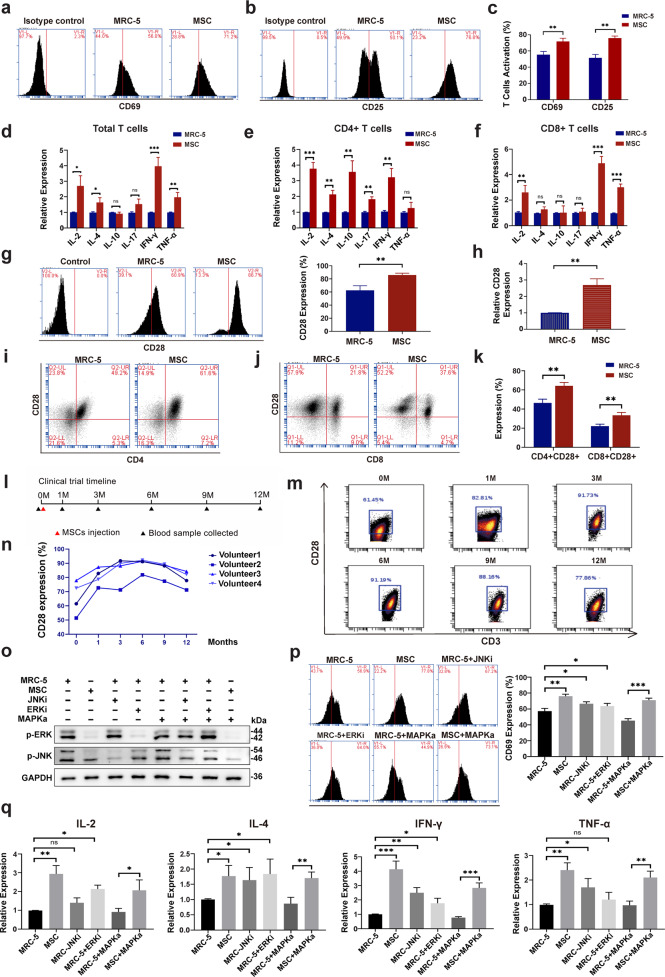
MSCs support immune function and promote costimulatory CD28 expression partly via MAPK-ERK/JNK signaling. **a** Representative flow cytometry results for activated PBMCs after co-culturing with MRC-5 or MSCs for 2 days. CD69 is an early-stage activation marker of T cells. **b** Representative flow cytometry results for activated PBMCs after co-culture with MRC-5 or MSCs for 5 days. CD25 is a mid-stage activation marker of T cells. **c** Summary histogram of T-cell activation markers CD69 and CD25. **d–f** Relative expression levels of RNAs encoding cytokines in total T cells (**d**), CD4^+^ T cells (**e**), and CD8^+^ T cells (**f**). **g** Representative flow cytometry results (left) and summary histogram (right) of CD28 expression on the surface of quiescent T cells co-cultured with MRC-5 or MSCs. **h** CD28 gene expression of T cells co-cultured with MRC-5 or MSCs by qRT-PCR. **i** Representative scatter diagram of CD28 expression on quiescent CD4^+^ T helper cells co-cultured with MRC-5 or MSCs. **j** Representative scatter diagram of CD28 expression on quiescent CD8^+^ cytotoxic T cells co-cultured with MRC-5 or MSCs. **k** Summary histogram of CD28 expression on CD4^+^/CD8^+^ quiescent T cells co-cultured with MRC-5 or MSCs. **l** Timeline of clinical trials of MSC infusion. **m** Representative scatter diagram of changes in CD28 expression on CD3^+^ T cells isolated from blood samples from volunteers over 12 months. **n** Line chart of changes in CD28 expression on CD3^+^ T cells isolated from blood samples from all four volunteers over 12 months. **o** Western blotting results of p-ERK and p-JNK of T cells co-cultured with MRC-5 or MSCs. **p** Representative flow cytometry results (left) and summary histogram (right) of CD69 expression on activated T cells co-cultured with MRC-5/MSCs with or without different signaling inhibitors or activators. **q** Relative expression of cytokines in T cells co-cultured with MRC-5/MSCs with or without different signaling inhibitors or activators. The data represent means ± SD. The *P* values were determined using the unpaired Student’s *t-*test. *P < 0.05, **P < 0.01, ***P < 0.001.

To study the mechanism by which MSCs modulate the immune function in different T cell subtypes, we co-cultured MSCs or MRC-5 (a fibroblast cell line as a control) with quiescent human PBMCs from healthy volunteers for 5 days. Flow cytometry detected CD69^+^ (an early-stage T cell activation marker) and CD25^+^ T cells (a mid-stage T cell activation marker) on days 2 and 5 after stimulation with polyhydroxyalkanoates, respectively. Compared with MRC-5, MSCs enhanced the T-cell activation in both the early (2 days) and middle stages (5 days) (Fig. 4a–c). In addition, T cell proliferation was also enhanced (Supplementary information, Fig. S5a, b). Five days of co-culture of PBMCs with MSCs increased the expressions of IL-2, IL-4, IFN-γ and TNF-α in the total T cell population, whereas the expression levels of IL-10 and IL-17 were not significantly altered (Fig. 4d; Supplementary information, Fig. S5c). Both mRNA and protein levels of IL-2, IL-4, IL-10, IL-17 and IFN-γ were upregulated in CD4^+^ T cells, whereas TNF-α was not significantly changed in these cells (Fig. 4e; Supplementary information, Fig. S5d). Moreover, IL-2, IFN-γ and TNF-α were upregulated in CD8^+^ cytotoxic T cells, but IL-4, IL-10 and IL-17 were not significantly changed (Fig. 4f; Supplementary information, Fig. S5e).

To observe the effect of MSCs on the CD28 molecule, PBMCs from a healthy donor over 65 years old, which had low baseline levels of CD28 expression, were co-cultured with MSCs. After co-culture with MSCs, PBMCs expressed higher levels of CD28 (Fig. 4g, h). Remarkably, higher CD28 expression was detected in both CD4^+^ and CD8^+^ T cells when separately co-cultured with MSCs (Fig. 4i–k). We next performed a small-scale pilot study of MSC treatment with four healthy volunteers (Supplementary information, Table S1). Blood samples were collected before treatment and at 1, 3, 6, 9 and 12 months after 4-day treatment, and T cell CD28 expression was detected by flow cytometry (Fig. 4l). CD28 expression increased continuously in T cells up to 6 months and then began to decline; nonetheless, the levels at 12 months were still higher than those at baseline (Fig. 4m). This trend was observed in all four volunteers (Fig. 4n). These results suggested that MSCs may be used to augment and maintain the percentage of CD28^+^ T cells in humans.

To explore the possible mechanism, we examined some key signaling molecules and found that the phosphorylation of ERK and JNK in T cells was downregulated after co-culture with MSCs. MSCs exerted a function similar to that of the inhibitors ERKi and JNKi, which inhibit the phosphorylation of ERK and JNK, respectively (Supplementary information, Fig. S5f). Insofar as MAPK activator can be used to activate ERK/JNK signaling, we added a JNK inhibitor (tanzisertib), ERK inhibitor (FR180204), or MAPK activator (anisomycin) to the co-culture system to confirm the contribution of MAPK-ERK/JNK signaling. Interestingly, JNKi and ERKi appeared to inhibit the phosphorylation of ERK and JNK, respectively. Moreover, MSCs could restrain the activation of the ERK/JNK signaling induced by MAPKa (Fig. 4o). Consistently, functional studies also revealed that both JNKi and ERKi promoted T cell activation and proliferation, whereas MAPKa inhibited the T cell activation and proliferation, which was reversed upon co-culture with MSCs (Fig. 4p; Supplementary information, Fig. S5g, h). Furthermore, JNKi, ERKi, and MAPKa altered the secretome of T cells (Fig. 4q). Both JNKi and ERKi increased the proportion of CD28^+^ T cells, whereas MAPKa had the opposite effect (Supplementary information, Fig. S5i). These findings indicated that MSCs can support T-cell functions by regulating MAPK-ERK/JNK signaling.

### MSCs promote the repair of lung damage through immune regulation in mice

**Fig. 5:**
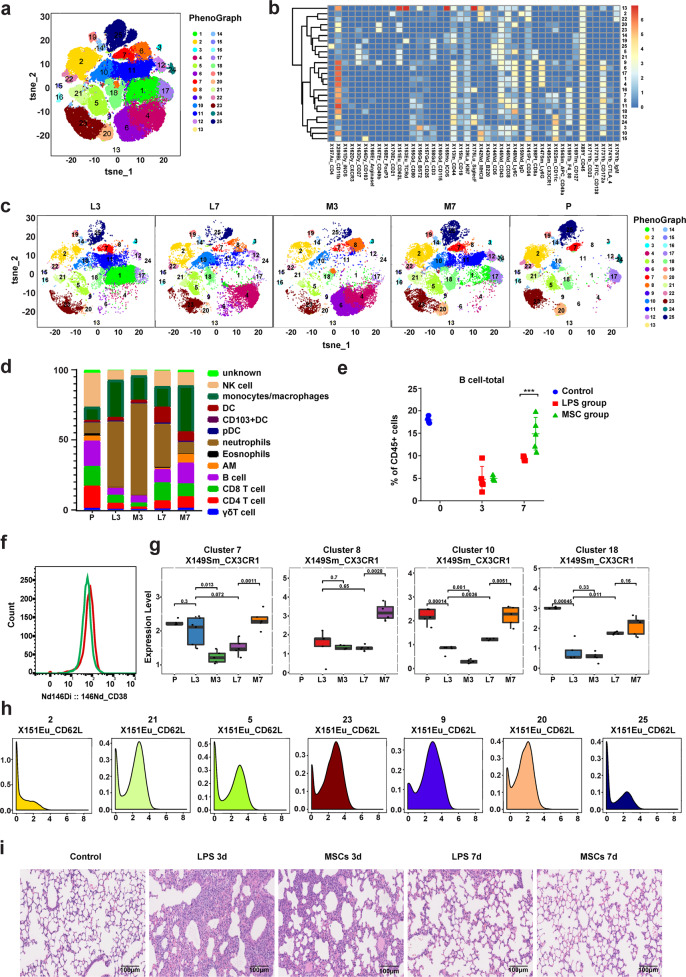
Mass-cytometry analysis of lung immune cells in LPS-treated and MSC-LPS-treated mice. **a** Identification of 25 distinct clusters of lung immune cells using tSNE and PhenoGraph. **b** Heatmap of the normalized expression of markers of various lung immune cell types. **c** viSNE map of lung immune cells. **d** Composition of lung immune cells in LPS-treated and MSC-LPS-treated mice. **e** Percentage of total B cells over time in LPS-treated and MSC-LPS-treated mouse lungs. **f** Histogram of CD38 expression in B cells. The red line represents the MSC-LPS-treated group, and the green line represents the LPS-treated group. **g** CX3CR1 expression on distinct cell clusters. **h** CD62L expression on distinct cells clusters. **i** Lung morphology in LPS-treated and MSC-LPS-treated mice. The data represent the means ± SD. L3, LPS-treated group on day 3; L7, LPS-treated group on day 7; M3, MSC-LPS-treated group on day 3; M7, MSC-LPS-treated group on day 7; P, control, PBS group. The *P* values were determined using the unpaired Student’s *t-*test. ***P < 0.001.

The lipopolysaccharide (LPS)-induced acute lung injury model demonstrates a severe immune response characterized by diffuse interstitial and alveolar edema, inflammatory cell infiltration, and the release of proinflammatory factors, which are similar to the symptoms of COVID-19 caused by the rapid replication of SARS-CoV-2 in the lungs.^30,31^ We next investigated the effect of MSCs on lung injury repair. We employed mass-cytometry to examine the alterations of lung immune cells in C57BL/6 mice after acute lung-injury induced by LPS. The mass-cytometry findings were analyzed by the algorithms t-distributed stochastic neighbor embedding (tSNE) and PhenoGraph. A total of 25 clusters were identified (Fig. 5a), and the expression levels of markers for each cluster were shown in a heatmap (Fig. 5b). Based on the expression levels of CD45, CD3, TCRd, CD4, CD8a, CD19, CD49b, CD11b, Siglec F, CD11c, F4/80, Ly6C, Ly6G, BST2, and CD103 (Supplementary information, Fig. S6a), 12 major cell categories were identified from 25 clusters, including γδ T cells, CD4^+^ T cells, CD8^+^ T cells, B cells, NK cells, alveolar macrophages, eosinophils, neutrophils, pDCs, CD103^+^ DCs, DCs and monocytes/macrophages (Supplementary information, Table S4). We further analyzed the fingerprint-like signatures and found that the control, LPS-treated, and MSC-LPS-treated lungs displayed different immune signatures (Fig. 5c). The lung immune niches of MSC-LPS-treated mice were mainly composed of myeloid cells (primarily neutrophils) on day 3, and monocytes/macrophages on day 7. The B cells, T cells, and alveolar macrophages (AMs) were significantly increased on day 7 compared with those on day 3 (Fig. 5d). These alterations of immune cells indicated that MSC treatment can induce immune responses after injury that are specific to certain immune cell types.

We next partitioned the lung B cells into two subsets: IgM^+^IgD^−^ B cells (cluster 22) and IgM^+^IgD^+^ B cells (cluster 2). The proportion of IgM^+^IgD^−^ B cells was significantly decreased on day 3, whereas the proportions of the total B cells and IgM^+^IgD^+^ B cells were both significantly increased on day 7 (Fig. 5e; Supplementary information, Fig. S6b). This is partially consistent with our previous hypothesis that the migration of some B cells to the damaged lungs may be responsible for the reduced B cell count in all PBMC in COVID-19 patients. Our results also demonstrated that these two B cell subsets may have different roles after MSC treatment. Importantly, CD38 expression on B cells was increased in MSC-LPS-treated lungs on day 7 (Fig. 5f), indicating that MSCs may facilitate B cell activation. The expression of the chemokine receptor CX3CR1, which regulates immune cell migration, was decreased on DCs and monocytes/macrophages on day 3 after MSC treatment (cluster 7 and 10), but it was increased (cluster 7, 8 and 10) on day 7 (Fig. 5g). Additionally, CD62L (also known as L-selectin) was highly expressed on B, T, neutrophil and NK cells (clusters 2, 5, 9, 20, 21, 23 and 25) (Fig. 5h). Histological assessment of the lungs after LPS administration revealed extensive interstitial infiltration by neutrophils and macrophages, and MSC treatment significantly reduced this infiltration (Fig. 5i), confirming that MSCs can regulate the immune response in mice with acute lung injury.

### MSCs upregulate integrin signaling in immune cells in COVID-19 patients and suppress NET release and venous thrombosis in mice

**Fig. 6:**
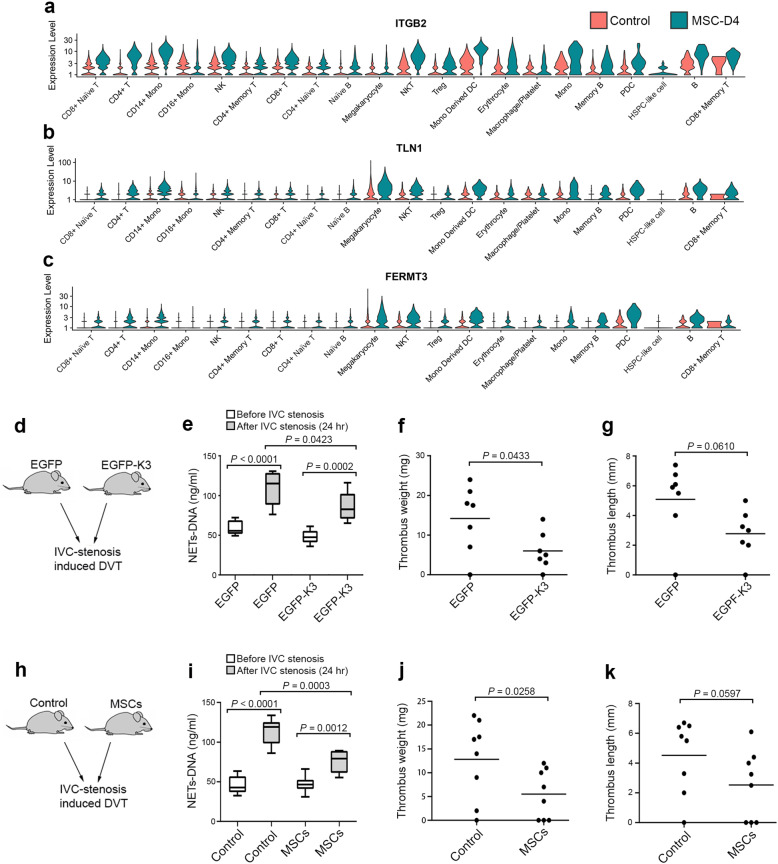
MSCs modulate inflammatory responses by enhancing integrin signaling and suppressing NET release and DVT in vivo. **a–c** Differential gene expression of β2-integrin (ITGB2) and the integrin activators talin-1 (TLN1) and kindlin-3 (FERMT3) in different leukocyte subpopulations of PBMCs isolated from control- or MSC-treated COVID-19 patients (MSC-D4). **d** DVT was induced by partially ligating IVC (IVC stenosis) in two groups of mice, one exogenously expressing EGFP and the other expressing EGFP-kindlin-3 (EGFP-K3) in bone-marrow cells with WT background. **e** Plasma NETs-DNA levels in mice (EGFP group and EGFP-K3 group) before and after IVC stenosis were measured using the Sytox Green assay. **f**, **g** Thrombi formed in the ligated IVC were collected from EGFP and EGFP-K3 mice. Weight (**f**) and length (**g**) were measured for each thrombus. **h** DVT was induced by IVC stenosis in MSC-treated mice and placebo-treated control mice. **i** Plasma NETs-DNA levels in control and MSC-treated mice before and after IVC stenosis were measured using the Sytox Green assay. **j**, **k** Thrombi that formed in the ligated IVC were collected from the control and MSC-treated mice. Weight (**j**) and length (**k**) were measured for each thrombus. Control, MSC-untreated controls. The data represent the means ± SD. The *P* values were determined using the unpaired Student’s *t-*test.

The β2-integrin family plays a key role in immune responses by mediating immune cell adhesion and transmigration to the sites of infection.^32^ Upon pathogen challenge, β2-integrins on leukocytes can be activated by two cytoplasmic integrin activators, talin-1 and kindlin-3.^33^ The deficiency of kindlin-3 in humans causes leukocyte adhesion deficiency III, characterized by recurrent infections and severe bleeding.^34,35^ To evaluate the effect of MSC treatment on integrin signaling in immune cells of COVID-19 patients, we compared the expression levels of integrin signaling molecules in PBMCs isolated from COVID-19 patients with or without MSC treatment based on scRNA-seq data. Fig. 6a–c show the differential expression levels of integrin β2 subunit, talin-1, and kindlin-3, which were substantially upregulated in both innate immune cells and lymphocytes in MSC-treated COVID-19 patients compared with those in control samples. These results suggest that MSC treatment may enhance the antiviral immune responses in COVID-19 patients by promoting integrin-mediated immune cell recruitment.

As an essential integrin activator,^18^ the presence of kindlin-3 in neutrophils also negatively regulates NET release, which can subsequently suppress venous thrombosis in mice, as demonstrated in our previous studies.^19,36 ^Therefore, we hypothesized that the elevated expression of kindlin-3 in myeloid cells found in MSC-treated COVID-19 patients might help restrict NET release and thereby reduce the risk of venous thrombosis. To test this hypothesis, we generated mice exogenously expressing EGFP-kindlin-3 in bone-marrow hematopoietic cells as well as mice exogenously expressing EGFP alone as the control. We employed the inferior vena cava (IVC) stenosis model by partially ligating the IVC to trigger deep vein thrombosis in these mice, as described by von Brühl et al.^37,38^ As shown in Fig. 6d, e, IVC stenosis substantially increased the levels of plasma NETs-DNA in mice. Importantly, the levels of plasma NETs-DNA in EGFP-kindlin-3 mice were significantly lower than those in EGFP mice, thus verifying that upregulation of kindlin-3 in hematopoietic cells can effectively suppress NET release in mice. As expected, venous thrombosis was significantly reduced in EGFP-kindlin-3 mice compared with EGFP mice (Fig. 6f, g). Importantly, MSC treatment suppressed both NET release and venous thrombosis in mice (Fig. 6h–k). Taken together, these results suggest that upregulation of kindlin-3 in bone marrow hematopoietic cells may serve as one of the mechanisms by which MSCs improve the outcome in COVID-19 patients.

## Discussion

**Fig. 7:**
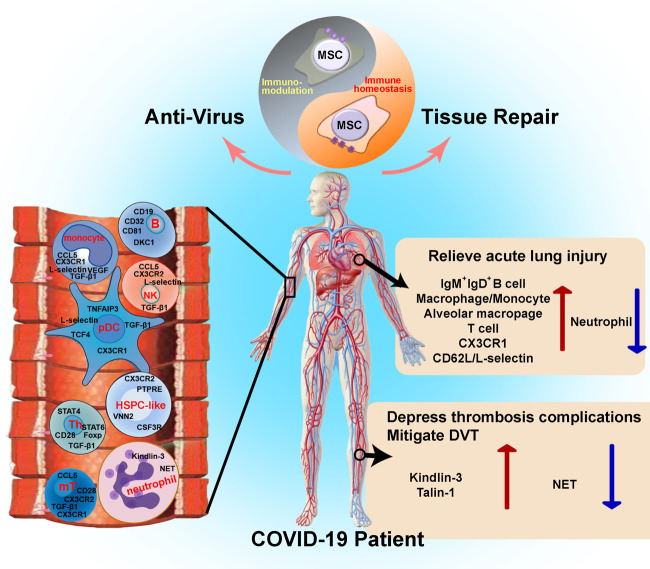
MSCs improve the prognosis of COVID-19 patients by modulating the immune esponse, promoting tissue repair, and suppressing NET release. MSCs orchestrate immunomodulatory functions in two main ways to restore a harmonious homeostasis of the immune microenvironment and promoting immune system recovery in COVID-19 patients. On the one hand, the treatment of patients with MSCs (1) induced mobilization of COVID-19 patient-derived VNN2^+^ HSPC-like cells to the peripheral blood of patients via upregulation of CSF3R and PTPRE; (2) induced upregulation of chemotaxis-related genes (CCL5, CXCR2, CX3CR1, and L-selectin) in activated monocytes, NK cells, pDCs, and memory T cells; (3) supported the function of T cells (upregulation of CD28) through MAPK-ERK/JNK signaling; and (4) promoted the differentiation of CD4^+^ T cells into Th cells (upregulation of CD28, CD40L, IL12R, STAT4 and STAT6) to assist in B cell activation (upregulation of CD19 and CD81). On the other hand, MSCs also inhibit the overactivation of immune cells and their immune response in patients, characterized by promoted immunomodulatory functions of pDC, and increased TGF-β1 in various immune cells, upregulation of FOXP3 in Th cells. Concordantly, MSC treatment induced novel immune responses and facilitated IgM^+^IgD^+^ B cell activation to promote repair of damaged lung tissue. Finally, MSC treatment reduced the production of neutrophil extracellular traps (NETs) in COVID-19 patients by upregulating kindlin-3 expression in immune cells to reduce the risk of immunothrombosis. mT, memory T cells.

Our results provide multiple lines of evidence demonstrating the potential of MSC infusion to improve the clinical outcomes of COVID-19 patients by modulating immunity, inhibition of NET release, elevation of plasma antibodies against SARS-CoV-2, and promoting lung injury repair (Fig. 7). Collectively, and together with our previous study,^11^ these findings suggest that MSCs are safe and efficacious for treating COVID-19. Moreover, this study suggested that MSCs can improve the outcome of patients with severe/critical symptoms more significantly, compared to that of the common/mild patients.

SARS-CoV-2 can attack the vast majority of cells in the body, causing damage to multiple tissues and organs.^36,39^ Moreover, COVID-19 patients have characteristic hyperinflammation and immune-function disorders.^40–42^ In this regard, MSCs reportedly can have powerful effects on the regulation of immune function and can reduce inflammation and consequent fibrosis in vivo.^43–45^ Increased plasma CRP and cytokines were documented for COVID-19 patients in our current trial as well as in other reports.^43–45 ^Our observation of reduced levels of proinflammatory mediators after MSC treatment suggests the remarkable ability of these cells to suppress inflammation and promote lung repair. MSC therapies have been shown to improve the survival of patients suffering from H7N9 influenza in both preclinical and clinical studies.^46^

Our scRNA-seq analysis revealed that a subpopulation of PBMCs isolated from MSC-treated patients expressed CSF3R and PTPRE, and these factors promote HSC mobilization, as reported by Greenbaum et al.^47^ and Bendall et al.^48^ Intriguingly, VNN2 — which is critical for HSC function during human embryonic period — was highly expressed in cluster 33. The fact that VNN2 can be used to distinguish a population of self-renewing HSCs allows these cells to be tracked in multiple developmental niches. A loss of VNN2 severely compromises HSC engraftment/reconstitution in vivo.^21^ Higher proportions of CD16^+^ monocytes after MSC infusion were found in the COVID-19 patients and similarly increased monocytes/macrophages were also found in MSC-LPS-treated mice with lung injury. These alterations of immune cells indicated that MSC treatment can induce immune responses after injury that are specific to certain immune cell types. Thus, we propose that the treatment of COVID-19 patients with MSCs creates a temporary extramedullary niche that supports the self-renewal and differentiation of HSCs in vivo for the benefit of regulating the inflammatory response and tissue repair.

We identified three additional molecules involved in chemotaxis — CD302, HMGB1 and L-selectin. The expression of these molecules is associated with peripheral blood cell migration and inflammation.^49–51^ We found that the CX3CR1 was highly expressed on DCs and monocytes/macrophages of MSC-treated patients, indicating that the migration of DCs and monocytes/macrophages might be mediated by CX3CR1. As reported by Zhang et al.^52^, CX3CL1 can mediate monocyte adhesion in the lung vasculature as well as the induction of VEGFA via its receptor CX3CR1 on monocytes. Altogether, our findings suggest that CX3CR1 and CD62L help facilitate the positive effects of MSC treatment on acute lung injury in mice. We found that MSC treatment promoted the expression of chemotaxis-related genes, which have been posited to participate in the recruitment of immune cells from peripheral blood to inflammatory sites to help restore the function of damaged organs.

Previous studies showed that telomerase activation in lymphocytes, especially T and B cells, is essential for their replicative capacity.^53^ The upregulation of genes involved in telomerase assembly and maturation may be a crucial step in MSC-mediated lymphocyte development, activation, immune maintenance, and tissue homeostasis in response to SARS-CoV-2 infection, because telomeres are shorter in virus-infected cells of COVID-19 patients with severe disease.^6,7^

MSCs facilitate the activation and subgroup changes of B cells, which may have promoted the production of antibodies specific for SARS-CoV-2 to facilitate recovery. It is widely accepted that an overactive immune response can have serious consequences in COVID-19 patients, such as an autoimmune response and/or cytokine storm, which could be prevented by regulating BCR signaling by increasing the expression levels of coinhibitory receptors.^41^ We found that MSCs were proficient at modulating B-cell activation to a level sufficient to combat COVID-19 by upregulating both costimulatory and coinhibitory receptors in patients’ B cells, which has not been demonstrated by other immunomodulatory therapies. Meanwhile, we were the first to report that MSCs can induce pDC differentiation,^54^ and we confirmed that MSCs are powerful immunoregulatory stem cells that can affect Treg subgroups and pDCs.^55–57^ These results support that MSC treatment can regulate the immune function in COVID-19 patients.

MAPK family kinases play a complex role in T cell development, activation and differentiation.^58–60^ A new study showed that MAPK inhibition reprograms CD8^+^ T lymphocytes into memory cells with potent antitumor effects.^61^ Consistently, we analyzed the scRNA-seq data and found that 2-day MSC treatment increased memory T populations, especially the CD8^+^ memory T populations (Fig. 2c), indicating a transient proliferation effect. MAPK inhibitors can coordinate with PD-L1 checkpoint blockers, to promote the immune function of T cells.^62^ Here, our results found that MSC treatment promotes T cell activation by restraining the MAPK-ERK/JNK pathway. These studies suggest that the complex functions of the MAPK pathway on T cells might be context-dependent.

Pneumonia can be induced by aseptic inflammation (such as LPS) or live pathogens (such as SARS-CoV-2), which is manifested as infiltration of inflammatory cells and inflammatory factors in the microenvironment of lung tissue, causing lung tissue damage. Therefore, clearing lung inflammation and regulating immune cell components in the injured tissue are important ways to repair lung injury.^63–65^ We found that the lung damage was mitigated by the treatment of MSCs, and the inflammatory cell infiltration was greatly reduced. BMSCs reprogramed monocytes and macrophages from septic lungs, producing greater amounts of IL-10, less TNF-α and IL-6, and prevented neutrophils from migrating into tissues and caused oxidative damage, thus mitigating lung damage.^66^ Our previous study also showed that MSCs could induce a novel Jagged-2-dependent regulatory DC population and promoted differentiation of regulatory DCs.^56,67^ These alterations of immune cells indicated that MSC treatment can induce immune responses after injury that are specific to certain immune cell types. Thus, we propose that the treatment of COVID-19 patients with MSCs creates a temporary extramedullary niche that supports the repopulation of tissue stem/progenitor cells in vivo for the benefit of regulating the inflammatory response and tissue repair.

Recent studies have established a robust correlation between the production of plasma NETs and respiratory illness severity in COVID-19 patients.^13,15,68,69^ Consistently, elevated neutrophil counts have been observed in patients with advanced COVID-19.^70,71^ As NETs can immobilize and degrade invading pathogens including viruses,^72^ increased NET release from neutrophils may help contain SARS-CoV-2 infection. However, excessive formation of NETs can also increase COVID-19 severity by contributing to immunothrombosis.^73^ Mechanistically, some pro-thrombotic factors carried by NETs, such as tissue factor and FXII, may directly contribute to triggering thrombosis.^37,74^ Therefore, the application of therapeutic strategies targeting NETs may help reduce the mortality associated with severe COVID-19. We found that MSC treatment can reduce plasma NETs-DNA levels in COVID-19 patients. Importantly, we observed that MSC treatment can upregulate both β2-integrins and essential integrin activators, talin-1 and kindlin-3, in immune cells of patients. Although the enhanced integrin activation in immune cells can potentially facilitate their recruitment and responses, elevated levels of kindlin-3 in neutrophils may also suppress NET release, as we previously reported.^18,35^ As expected, the efficacy of MSC treatment in attenuating deep venous thrombosis was verified in mice. These findings provide significant evidence to support the role of MSCs in mediating the immunomodulation in COVID-19 patients.

In summary, our results demonstrate that MSCs can contribute to the treatment of COVID-19 by regulating the immune microenvironment (Fig. 7). MSCs play various important roles in maintaining homeostasis, immune regulation and reconstitution, and tissue repair, which could enhance the efficacy of clinical treatment. Further studies are required to determine how MSCs modulate cellular and signaling networks in response to microenvironmental cues in COVID-19.

## Materials and methods

### Study design

This clinical study was a randomized, single-blind, controlled trial. It was conducted and approved in Beijing YouAn Hospital, Capital Medical University (LL-2020-013-K), and Puren Hospital Affiliated with Wuhan University of Science and Technology (No. 2020-001). The study was registered with the Chinese Clinical Trial Registry (ChiCTR2000029990) and ClinicalTrials.gov (NCT04339660), and each enrolled patient provided written informed consent. The trial was conducted in accordance with the principles of the Declaration of Helsinki and the International Conference on Harmonization—Good Clinical Practice guidelines. The safety and efficacy data for MSC infusion were assessed for 28 days after MSC treatment.

### Patients

A total of 58 patients were enrolled in this study, with 29 in the MSC-treatment group and 29 in the placebo-control group. Both groups received standard treatment according to the China Novel Coronavirus Diagnostic Guidelines. All patients diagnosed with COVID-19 were eligible to join this clinical study if they fulfilled the following criteria: (1) age range 18–95; (2) confirmed as infected with SARS-CoV-2 by qRT-PCR; (3) confirmed as having common, severe, or critically severe COVID-19 according to the guidelines of the National Health Commission of China. Patients were ineligible if they were pregnant, had been diagnosed as having cancer, or were experiencing critical respiratory failure. All participants provided written informed consent for the collection of information. All patients agreed to the publication of their data.

### Cell preparation and transplantation

Clinical-grade human umbilical cord-derived MSCs were supplied free of charge by Qingdao Co-orient Watson Biotechnology Group Co., Ltd., and the Institute of Basic Medical Sciences, Chinese Academy of Medical Sciences. These MSCs were certified by the National Institutes for Food and Drug Control of China (authorization numbers: 2004L04792, 2006L01037, CXSB1900004, SH201905140). Prior to intravenous drip, MSCs were suspended in 100 mL normal saline, and the total number of transplanted cells was calculated to be 1 × 10^6^ cells per kilogram body weight. The MSCs were administered intravenously for ~40 min at 20–30 drops per minute.

### Preparation of single-cell suspension samples

The loaded cell number ranged from ~10,000 to ~30,000 per sample. Each cell pellet from the MSC culture was subjected to two rounds of resuspension in 1 mL PBS (containing 0.04% bovine serum albumin) followed by recentrifugation. Then, an appropriate volume of PBS was added to each cell precipitate to obtain a single-cell suspension with a cell density appropriate for each particular experiment. A wide-bore pipette tip was used for cell resuspension to minimize cell damage. Automated cytometry was used to determine the cell concentration. Each sample volume was calculated based on the optimal cell sampling concentration supplied by the 10X Genomics official website and the target capture number. If the calculated concentration was extremely high for the purpose of any particular experiment, we adjusted the liquid volume to achieve the appropriate cell concentration and then repeated the counting. Once the desired cell suspension was achieved, we immediately placed the samples on ice for subsequent study.

### LPS-induced acute lung-injury model in mice and treatment of LPS-induced mice with MSCs

Mice (6–8 weeks old) were anesthetized with 2% chloral hydrate. Intratracheal inhalation of 20 mg/kg LPS was performed to induce acute lung injury. MSCs were isolated from bone marrow of C57BL/6 mice and were cultured in MSC special complete medium. MSCs from passage 3 were used for further treatment through intratracheal inhalation.^75^ The control group underwent the same operation, with PBS instead of LPS and MSCs.

### Mouse model with overexpressed Kindlin-3 in hematopoietic cells

Sca1^+^ bone-marrow cells were isolated from wild-type (WT) C57BL/6 mice using a Sca1^+^ selection kit (Stemcell) and cultured in DMEM supplemented with 15% fetal bovine serum (FBS; Gibco, Grand Island, NY, USA), 20 ng/mL IL-3, 50 ng/mL IL-6, and 50 ng/mL stem-cell factor (SCF). The kindlin-3-coding sequence was inserted into lentiviral vector pLeGo-G2 to generate lentiviral particles expressing EGFP-fused kindlin-3, which were further used to transduce bone-marrow cells (MOI = 5). Lentiviral particles carrying empty pLeGo-G2 were used to express EGFP in bone-marrow cells as a control. EGFP-positive cells were sorted 2 days after transduction and were transplanted to lethally irradiated WT C57BL/6 recipient mice. After 8 weeks, deep venous thrombosis was assessed.

### Adipose-derived MSCs from mice and their isolation and transplantation

Adipose tissue-derived MSCs were isolated from the epididymis region of C57BL/6 male mice (8–10 weeks), as described by Pedrazza et al.^76^ Cells were passaged every 3 days by trypsinization when they reached ~70% confluency and were used for experiments at passages 3 or 4. In the MSC treatment group, mice were transplanted with 6 × 10^5^ MSCs in 100 µL PBS by tail vein injection; control mice received 100 µL PBS. After treatment, the level of deep venous thrombosis was assessed.

### IVC stenosis-induced deep venous thrombosis (DVT) model in mice

C57BL/6 mice were anesthetized by isoflurane-oxygen inhalation. A laparotomy was performed to expose the IVC, which was carefully separated from the attached tissues at an area below the renal veins and ligated over a spacer (5.0 monofilament polypropylene filament). The spacer was carefully removed after ligation to avoid complete vessel occlusion. In addition, back branches were either ligated or cauterized. The peritoneum and skin were immediately closed and sutured. Mice were sacrificed 24 h later, and the IVC tissues and blood samples were collected for further quantification.

### 10× genomics transcriptome library construction and sequencing

SPRIselect beads were used to purify the product. Adaptor ligation and SPRIselect purification. Index PCR and SPRIselect purification. A Qubit^®^ 3.0 Fluorometer (Life Technologies, CA, USA) was used to determine the library concentration. The 2100 High Sensitivity DNA Assay kit (Agilent Technologies, CA, USA) was used to determine the distribution of library product fragments. After library construction, Agilent 2100/LabChip GX Touch was used to determine the distribution of the fragment length of the library. In addition, quantitative PCR was used to quantify the effective concentration of the library, the target of which was > 10 nM. Once qualified, the library was sequenced on an Illumina HiSeq platform.

### SARS-CoV-2 antibody assay

The levels of antibodies against SARS-CoV-2 RBD, nucleocapsid/N antibody, or spike S1 + S2 extracellular domain antibody were measured in plasma samples using commercial kits (Sino Biological, Beijing, China). Briefly, 96-well plates were pre-coated with 100 ng of either the three aforementioned recombinant SARS-CoV-2 proteins overnight. The next day, plasma samples were diluted 1:200, 1:1000, or 1:5000 with PBS containing 0.5% Triton X-100 and 5% FBS; and these samples were added into wells of the 96-well plate. Then, horseradish peroxidase-conjugated goat anti-human IgG was added. Tetramethylbenzidine (TMB) substrate solution was used, and the optical density was measured at 450 nm.

### Isolation of PBMCs and co-culture with MSCs or MRC-5

Human PBMCs were isolated as described by Kong et al.^77^ Transwell plates (Corning) were used for the co-culture system. X-radiated MSCs or MRC-5 cells were seeded in the lower plates, whereas PBMCs were cultured in the upper chambers.^78^ The co-culture medium was composed of RPMI 1640 (Corning), 10% FBS, 1 mM glutamine, 0.1 mM β-mercaptoethanol, 1% non-essential amino acids (Sigma-Aldrich), and 5 ng/mL IL-2 (Preprotech).

### T cell activation assay

PBMCs were non-specifically activated with phytohemagglutinin (PHA) or specifically activated with an antibody against CD3. CD69 and CD25—markers of early-stage and mid-stage activated T cells, respectively — were detected after 2 days (CD69) or 5 days (CD25) by flow cytometry.

### CFSE proliferation assay

T cells were stained with CFSE living cell dye (Biolegend) for 30 min in darkness at 4 °C prior co-culture with MSCs. After 3 days, T cells were harvested for flow cytometry and analyzed using ModFit software. Cells with higher fluorescence were identified as the parent generation.

### MTS proliferation assay

Cells were placed into wells of several 96-well plates at 10,000 cells per well. MTS reagent (Promega) was added to each well of an individual plate at time 0 or on day 1, 3, 5 or 7. After 3 h incubation in a humidified atmosphere containing 5% CO_2_, the absorbance was measured at 490 nm. Proliferation curves were established according to relative MTS values from day 0 to day 7 using GraphPad Prism 8 software.

### Cytokine detection

The cytokines secreted by T cells were detected with a human cytokine/chemokine magnetic bead panel kit (Millipore, Billerica, MA, USA), also known as high-throughput ELISA. To detect the effect of MSCs on the production of cytokines or chemokines in the serum of enrolled patients, proinflammatory cytokines were measured using the Human Cytokine Factor Panel A Magnetic Bead Panel 96-Well Plate Assay (EMD Millipore, Billerica, MA USA) using Luminex^®^ 200^™^ with xPONENT^®^ software.

### Protein microarray analysis

Protein microarray analysis was performed by Wayen Biotechnology using the Full Moon CSP100 plus microarray analysis kit. A total of 304 proteins or phosphorylated proteins in the 16 signaling pathways were assayed.

### RNA isolation and qRT-PCR

Total RNA was extracted from cultured MSCs with TRIzol reagent (Invitrogen) and then quantified by spectrophotometry with a Nanodrop ND1000 instrument (Thermo Scientific). RNA was reverse transcribed with the Reverse Transcription kit (Takara). qRT-PCR was performed using the SYBR premix Ex Taq (Takara) using QuantStudio 3 (Applied Biosystems) in 10-µL reactions in triplicate. Data were analyzed with QuantStudio Design and analysis software (Applied Biosystems). GAPDH was used as an internal control. The primers used in this study were listed in Supplementary information, Table S2.

### Protein extraction and western blotting assay

Cells were washed twice with cold PBS. Total protein was extracted by RIPA lysis buffer accompanied with 1 mM phenylmethylsulfonyl fluoride along with a proteinase inhibitor cocktail (Beyotime) and phosphatase inhibitor cocktail (Yeasen) and then quantified using the BCA Protein Assay kit (Beyotime). Western blotting assay was performed as described.^55^ Extracts of soluble cellular protein (20 µg) were separated by 10% SDS-PAGE and then transferred to a 0.45 µm polyvinylidene difluoride membrane (Millipore). After blocking with 5% bovine serum albumin for 1 h at 25 °C, each membrane was incubated with specific primary antibodies (Proteintech or Cell Signaling Technology) at 4 °C overnight. On the next day, each membrane was incubated with an appropriate horseradish peroxidase-conjugated secondary antibody (Neobioscience) for 1 h at room temperature. Immunopositive bands were visualized via chemiluminescence (ECL reagent, Millipore) using a Model 4600 Chemiluminescence Imaging System (Tanon).

### Lung pathological examination

Lung specimens were fixed in 4% paraformaldehyde, embedded in paraffin, and sectioned to a thickness of 5 μm. Sections were stained with hematoxylin and eosin and then examined by light microscopy.

### Quantification of DNA in blood plasma

Blood samples were collected from COVID-19 patients or from model mice. Plasma DNA was quantified using the Sytox Green assay, as described previously.^18,19^ In brief, each plasma sample was diluted 1:10 in triplicate wells of a 96-well plate (total volume, 100 µL per well), and 10 µL of Sytox Green solution (10 µM) was added. After mixing, the fluorescence intensity was measured using a fluorescence plate reader (PerkinElmer). The DNA concentration in each sample was calculated based on a standard curve.

### Data processing and identification of differentially expressed genes (DEGs) and marker genes

scRNA-seq data were initially processed using the Cell Ranger (3.1.0) pipeline (https://support.10xgenomics.com/single-cell-gene-expression/software/pipelines/latest/using/count) with the hg19 human reference genome obtained from 10x Genomics (http://cf.10xgenomics.com/supp/cell-exp/refdata-cellranger-hg19-1.2.0.tar.gz). Briefly, first, the sequences in the FASTQ files for seven COVID-19 patient samples were aligned to hg19 with STAR software using the Cell Ranger ‘count’ module. Second, a feature-barcode matrix was generated from the Cell Ranger ‘count’ module to computationally analyze cell clusters. Seurat 3.1 (R package) was used for data filtration, scaling, integration, clustering, dimension reduction, differential expression analysis, and visualization. A total of 90,516 cells were collected, for which <200 or >5000 genes were differentially expressed and contained >10% mitochondrial genes; genes expressed in fewer than three cells were regarded as abnormal and were filtered out. The seven filtered gene-barcode matrices were first normalized using ‘LogNormalize’ methods, and the top 2000 variable genes were identified using the ‘vst’ method. These genes were then integrated using the ‘IntegrateData’ function in Seurat. After scaling the data, dimension reduction was performed using principal component analysis, cell clustering was performed using a graph-based method, and visualization was achieved with tSNE and UMAP (uniform manifold approximation and projection) considering the top 50 principal components in Seurat. Then, to identify the DEGs and marker genes under a particular condition, we selected the UMAP results as the final visualization of the 37 cell clusters in Seurat. The specific marker genes for each cluster were identified using the Seurat ‘FindMakers’ function. Specifically, the DEGs were identified by comparing cells in a particular cluster with all cells of the other 36 clusters. Then, the DEGs were selected as specific marker genes for that cluster, with the average expression in that cluster based on the LFC (log fold change) and *P* value. All marker genes within any particular cluster had to be in the top of the upregulated or downregulated genes in that cluster.

### Identification of cell types

Typically, cell types within a cluster can be directly identified based on canonical cell types using canonical marker genes that strongly indicate which clusters represent the corresponding cell types. For novel clusters or those clusters that lacked canonical marker genes, we used the DEGs as marker genes for cell type identification. We compared gene expression in cells belonging to each cluster with that in all other cells to obtain markers for each cluster. Finally, genes were ranked based on their average expression (largest to smallest) or *P* values (smallest to largest). The top-ranked genes were considered as marker genes for inferring the cell types. To ensure the accuracy of cell-type identification, we combined canonical marker genes and top-ranked DEGs to identify cell types for all 37 clusters using SCSA tools.

### Flow cytometry analysis

Flow cytometry was performed as described by Zhang et al.^67^ In brief, T cells were harvested from transwell chambers and were washed twice with culture medium before incubating with fluorescein-conjugated antibodies or isotype antibodies (Biolegend) in culture medium for 15 min at 4 °C in the dark. Cells were then washed twice prior to analysis with an Accuri C6 flow cytometer (BD Biosciences) and quantified with CFlow Plus software and Flowjo software (BD Biosciences).

### SNP analysis

A total of 16 subgroups of CD4^+^ T cells, B cells, and bone-marrow stromal cells in different periods of different samples were selected, and cells of the same subgroup were combined into a single sample. According to the barcode information for different subgroups, the mapping data for 16 cell populations were extracted through pysam. After recalibrating the data, GATK (version v3.7-0) and HaplotypeCaller were used for SNP/INDEL analysis and for further filtering of polymorphic sites. The SNP filtering parameters were DP < 4 || QD < 2.0 || FS > 60.0 || MQ < 40.0 || MQRankSum < −12.5 || ReadPosRankSum < −8.0. The INDEL filtering parameters were DP < 4 || QD < 2.0 || FS > 200.0 || ReadPosRankSum < −20.0. The polymorphic sites were filtered with PASS tags, and plink was used to remove missing sites. Furthermore, flashpca was used for principal component analysis, which finally yielded the two-dimensional coordinates of 16 samples. A joint calling strategy was used to call the most confident SNPs.^79^ The heterozygous or homozygous status for each SNP was then determined based on the reads in each sample.

### Mass-cytometry and data analysis

For each mouse, both lungs were removed and digested to yield a single-cell suspension using a mouse Lung Dissociation kit (Miltenyi Biotec, CA, USA).^31^ Mouse CD45-conjugated beads were added to collect CD45^+^ immune cells. A mass-cytometry panel of metal isotope-tagged antibodies was used to evaluate CD45^+^ cells in mouse lungs. Data were collected using a Helios system (Fluidigm Sciences, CA, USA) and analyzed using R version R 3.6.1. Data were normalized, transformed, and clustered with cytofAcsih and PhenoGraph. The t-distributed stochastic neighbor embedding method was applied to visualize the mass-cytometry data. Data presentation was implemented with ggplot2 (R package).

### GO analysis

GO enrichment analyses were based on significantly differentially expressed genes using clusterProfiler 3.14.3 (R package).

### Statistical analysis

Clinical outcomes of COVID-19 patients based on computed tomography scans on days 7, 14, and 21, as well as mortality by day 28, were analyzed and compared by the *χ*^2^ test or Fisher exact test. The cumulative percentage of patients who experienced remission of clinical symptoms and time from enrollment in the study to discharge were presented in a Kaplan–Meier plot and compared using the log-rank test. Hazard ratios with corresponding 95% confidence intervals were calculated using the Mantel–Haenszel approach. Mean levels of plasma CRP and inflammatory cytokines were compared using unpaired or paired Student’s *t-*test for normally distributed, continuous variables, and the median and range and Wilcoxon test were given for variables that were not normally distributed. The *χ*^2^ test or Fisher’s exact test was used to analyze categorical data, and categorical variables were expressed as a number (%). Continuous variables were expressed as the median (interquartile range), compared using the unpaired Student’s *t-*test, and reported as point estimates and 95% confidence intervals. Safety analyses were based on each patient’s specific treatment. For clinical laboratory data, the statistical significance of differences between groups was analyzed using the Student’s *t-*test or analysis of variance. The data represent the means ± SEM. All statistical analyses were performed using the Statistical Package for the Social Sciences version 20 (SPSS Inc., Chicago, Ill) or Prism 6 software (GraphPad, San Diego, CA). *P* values of < 0.05 (two-tailed) were considered statistically significant.

Gene levels were analyzed through Seurat analysis during RNA-seq analysis. DEG analysis was performed by the Wilcox test, and the DEGs were obtained when LFC ≥ 1 and the *P* value was < 0.05. Therefore, the gene level changes were considered statistically significant.

## Supplementary information

Supplementary Figure S1

Supplementary Figure S2

Supplementary Figure S3

Supplementary Figure S4

Supplementary Figure S5

Supplementary Figure S6

Supplementary Table S1

Supplementary Table S2

Supplementary Table S3

Supplementary Table S4

Supplementary Materials and Methods
