# Supplementary material for: Mesenchymal stem cell treatment improves outcome of COVID-19 patients via multiple immunomodulatory mechanisms
Source: Cell Res. 2021 Oct 26;31(12):1244–62. doi: 10.1038/s41422-021-00573-y (PMC8546390; doi:10.1038/s41422-021-00573-y)
Supplement: Supplementary file 1 — Supplementary Figure S1 [file 41422_2021_573_MOESM1_ESM.pdf]

Fig. S1

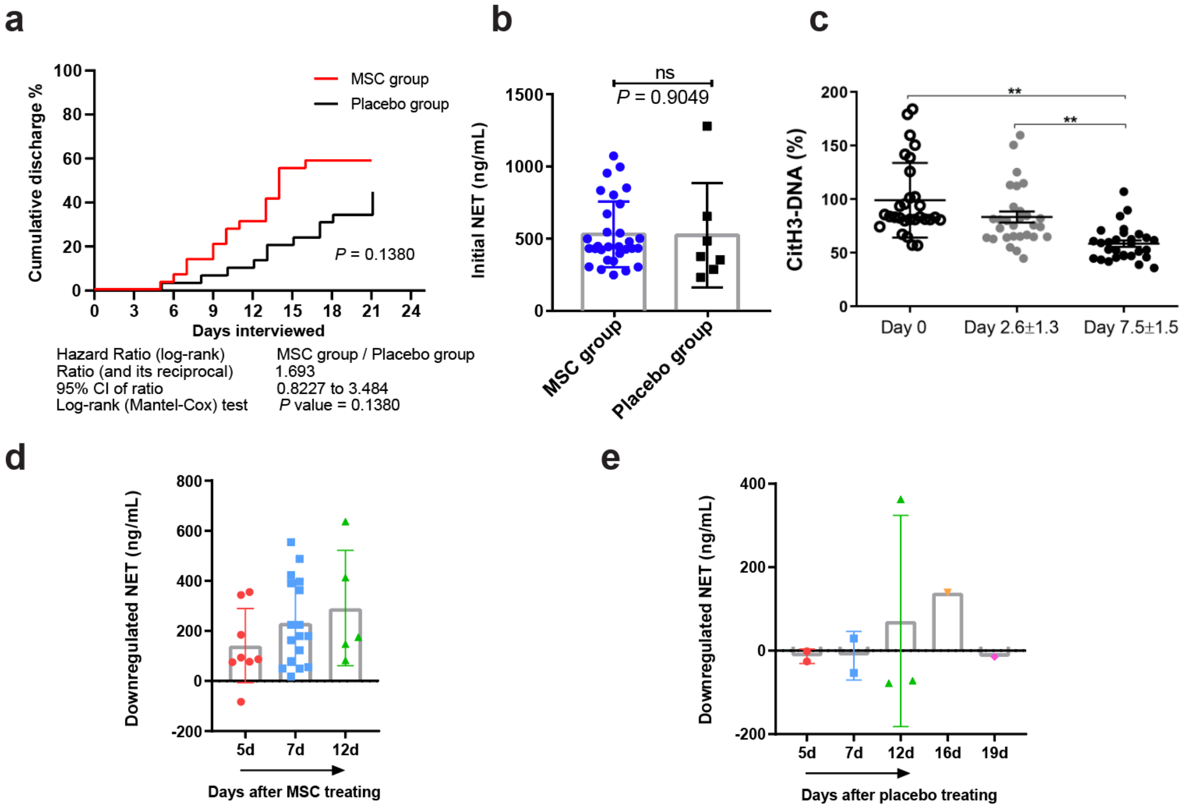

Fig. S1 NET-DNA Levels in COVID-19 Patients in the MSC-Treated and Placebo-Treated Groups. a Cumulative discharge rate of the two groups. **b** The initial NET levels of COVID-19 patients in the MSC-treated and placebo-treated groups. **c** Plasma CitH3-DNA levels in MSC-treated patients. Blood samples were collected from COVID-19 patients after MSC treatment at different time points, as indicated. The levels of the CitH3-DNA complex in plasma were measured by ELISA and expressed as percent of Day 0 controls. $^{**}P < 0.01$. **d-e** Plasma NET-DNA levels changed over time in both MSC-treated patients (**d**; n = 22) and placebo-treated patients (**e**; n = 7). The data represent the mean ± SD. The *P* values were determined using the unpaired Student's t-test OR t-test, as shown in the graph. $^{**}P < 0.01$. Related to Fig. 1.
