# Supplementary material for: Mesenchymal stem cell treatment improves outcome of COVID-19 patients via multiple immunomodulatory mechanisms
Source: Cell Res. 2021 Oct 26;31(12):1244–62. doi: 10.1038/s41422-021-00573-y (PMC8546390; doi:10.1038/s41422-021-00573-y)
Supplement: Supplementary file 2 — Supplementary Figure S2 [file 41422_2021_573_MOESM2_ESM.pdf]

Fig. S2

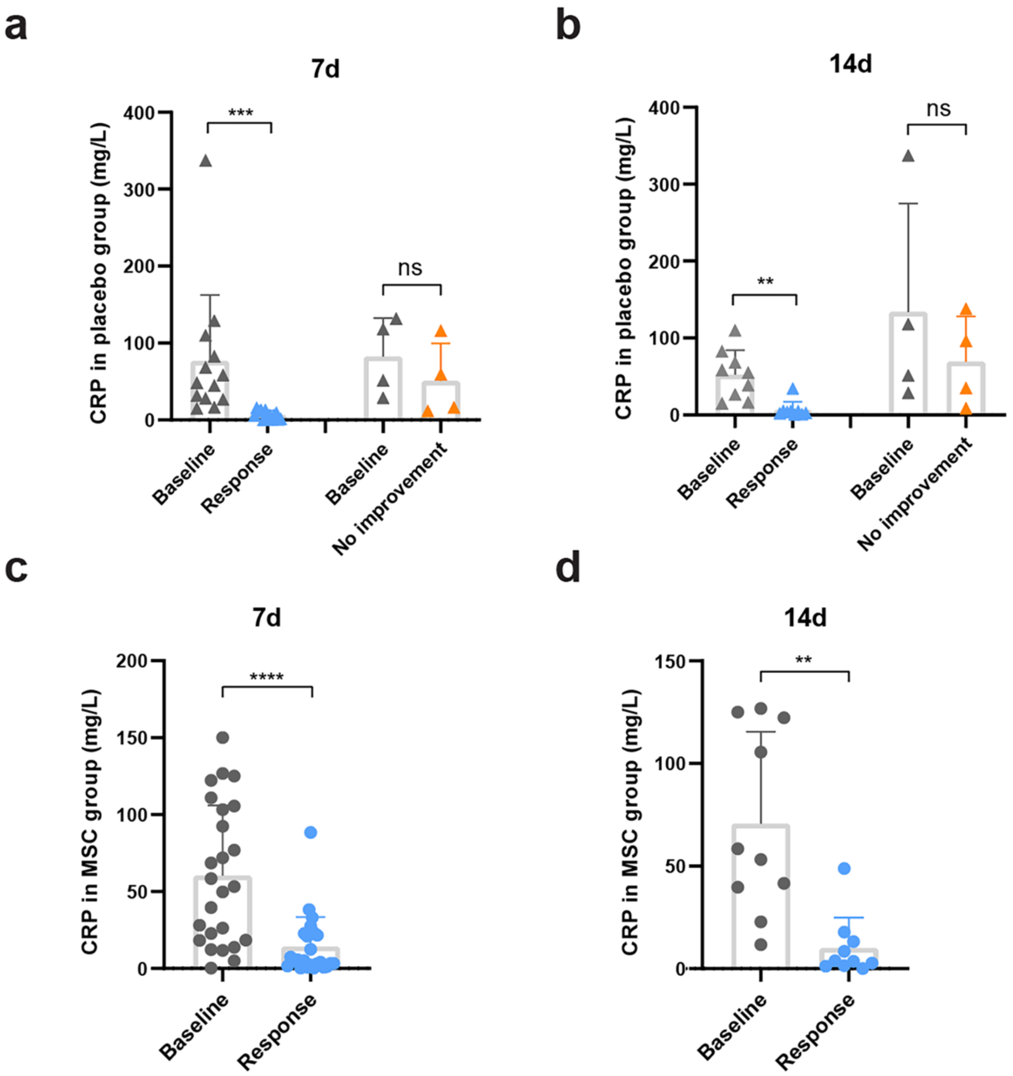

Fig. S2 The Changes in Plasma CRP with the Treatment Effects were Evaluated within the Two Groups. CRP levels changes in symptom response, or no improvement patients **(a)** by day 7 (n = 17, P = 0.0002) or **(b)** day 14 (n = 13, P = 0.0078) in the placebo group. CRP levels changes in symptom response patients **(c)** by day 7 (n = 25, P < 0.0001) and **(d)** day 14 (n = 10, P = 0.0039) in the MSC group. The data represent the mean ± SD. d, days. CRP, C-reactive protein. Response, remission or improvement. The P values were determined using the paired Student t-test or Wilcoxon test. Related to Fig. 1.
