# Supplementary material for: Mesenchymal stem cell treatment improves outcome of COVID-19 patients via multiple immunomodulatory mechanisms
Source: Cell Res. 2021 Oct 26;31(12):1244–62. doi: 10.1038/s41422-021-00573-y (PMC8546390; doi:10.1038/s41422-021-00573-y)
Supplement: Supplementary file 3 — Supplementary Figure S3 [file 41422_2021_573_MOESM3_ESM.pdf]

Fig. S3

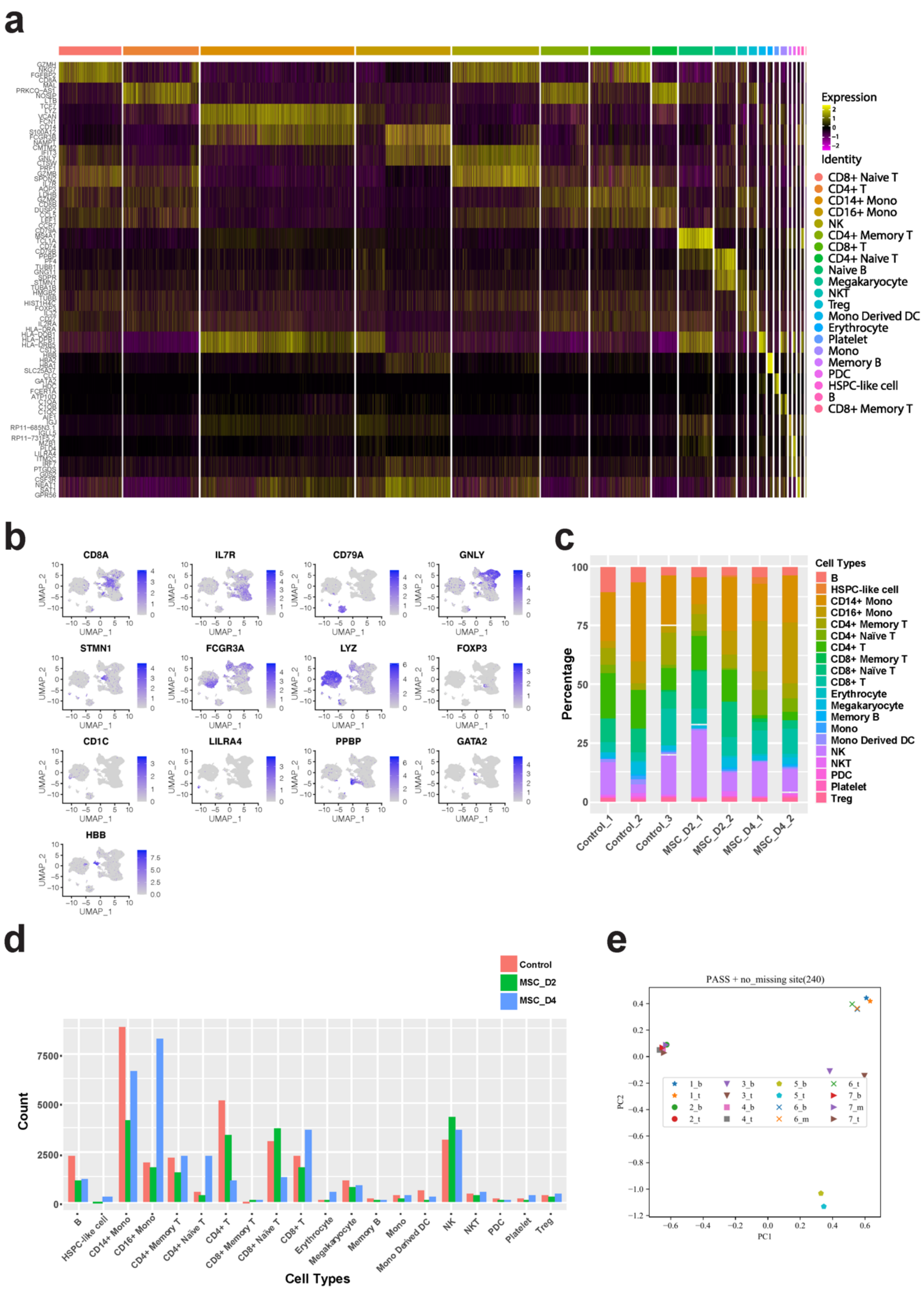
a
Expression
Identity
CD8+ Naive T
CD4+ T
CD14+ Mono
CD16+ Mono
NK
CD4+ Memory T
CD8+ T
CD4+ Naive T
Naive B
Megakaryocyte
NKT
Treg
Mono Derived DC
Erythrocyte
Platelet
Mono
Memory B
PDC
HSPC-like cell
B
CD8+ Memory T
b
CD8A
IL7R
CD79A
GNLY
STMN1
FCGR3A
LYZ
FOXP3
CD1C
LILRA4
PPBP
GATA2
HBB
UMAP_1
UMAP_2
c
Percentage
Cell Types
B
HSPC-like cell
CD14+ Mono
CD16+ Mono
CD4+ Memory T
CD4+ Naive T
CD4+ T
CD8+ Memory T
CD8+ Naive T
CD8+ T
Erythrocyte
Megakaryocyte
Memory B
Mono
Mono Derived DC
NK
NKT
PDC
Platelet
Treg
Control_1
Control_2
Control_3
MSC_D2_1
MSC_D2_2
MSC_D4_1
MSC_D4_2
d
Control
MSC_D2
MSC_D4
Count
Cell Types
e
PASS + no_missing site(240)
PC1
PC2

Fig. S3 Clustering Information for PBMCs from COVID-19 Patients. a Heatmaps for the top two most differentially expressed genes across 37 clusters. **b** UMAP projection of canonical markers of major cell types (n = 7). **c** The proportion of the major immune-cell types at a single sample. **d** Bar plot showing the counts of the major immune-cell types in samples among PBMCs from MSC-treated COVID-19 patients (MSC-D2 and MSC-D4) and MSC-untreated control samples. **e** Distribution of SNP principal components (PC) in $CD4^+$ T cells, B cells, and HSPC-like cells in seven samples. The cell clusters are named X_Y, where X represents the sample number (1, 2,...,6,7), and Y represents the cell type (t: T cells, b: B cells). Sample 2 was acquired from a patient before treatment, samples 4 and 7 were from the same patient after MSC treatment, and sample 5 was collected from a healthy control. Although SNP loci of the day 2 post-MSC treatment sample (sample 3) belonging to the third patient on PC2 differed from the loci of the control sample (sample 1) and the day 4 post-MSC treatment sample (sample 6), on PC1, the three samples were consistent with regard to SNP status. The two groups of HSPC-like cells (6_m and 7_m) formed on day 4 post-MSC treatment in two patients were mainly grouped with other two clusters (6_t, 6_b, and 7_t, 7_b). UMAP, Uniform manifold approximation and projection for dimension reduction; control, MSC-untreated controls. Related to Fig. 2.
