# Supplementary material for: Mesenchymal stem cell treatment improves outcome of COVID-19 patients via multiple immunomodulatory mechanisms
Source: Cell Res. 2021 Oct 26;31(12):1244–62. doi: 10.1038/s41422-021-00573-y (PMC8546390; doi:10.1038/s41422-021-00573-y)
Supplement: Supplementary file 4 — Supplementary Figure S4 [file 41422_2021_573_MOESM4_ESM.pdf]

Fig. S4

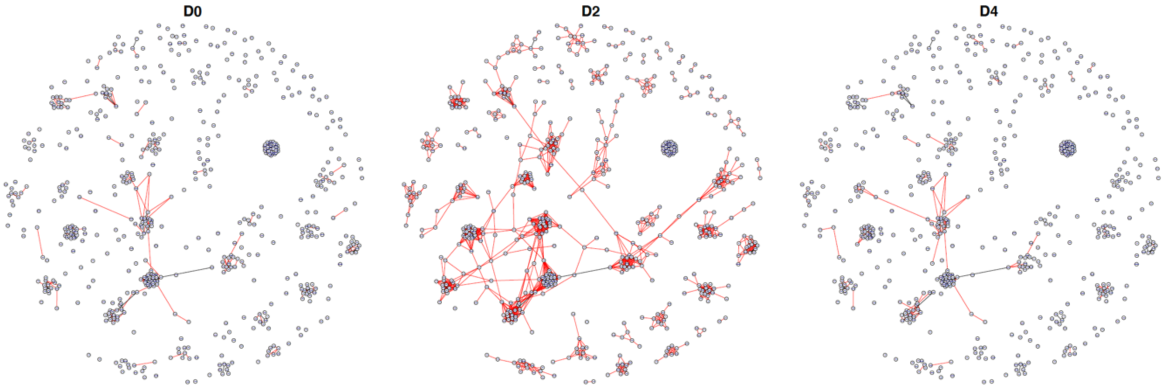

Fig. S4 Differential Co-Expression Networks in Monocytes of Patients with Good Prognosis on Days 0, 2 and 4. The black edges show co-expression pairs at three time points, and red edges show the changes in co-expression with time. For each cell type, we enriched the top 500 most highly expressed genes to yield 332 genes that interact with SARS-CoV-2 using the hypergeometric test. To identify the pathways most affected by MSC treatment, we performed differential co-expression analyses using R package DiffCorr and found that the network on day 2 was significantly rewired, with many activated interactions, and the network on day 4 was largely restored to that of day 0. Related to Fig. 2.
