# Supplementary material for: Mesenchymal stem cell treatment improves outcome of COVID-19 patients via multiple immunomodulatory mechanisms
Source: Cell Res. 2021 Oct 26;31(12):1244–62. doi: 10.1038/s41422-021-00573-y (PMC8546390; doi:10.1038/s41422-021-00573-y)
Supplement: Supplementary file 5 — Supplementary Figure S5 [file 41422_2021_573_MOESM5_ESM.pdf]

Fig. S5

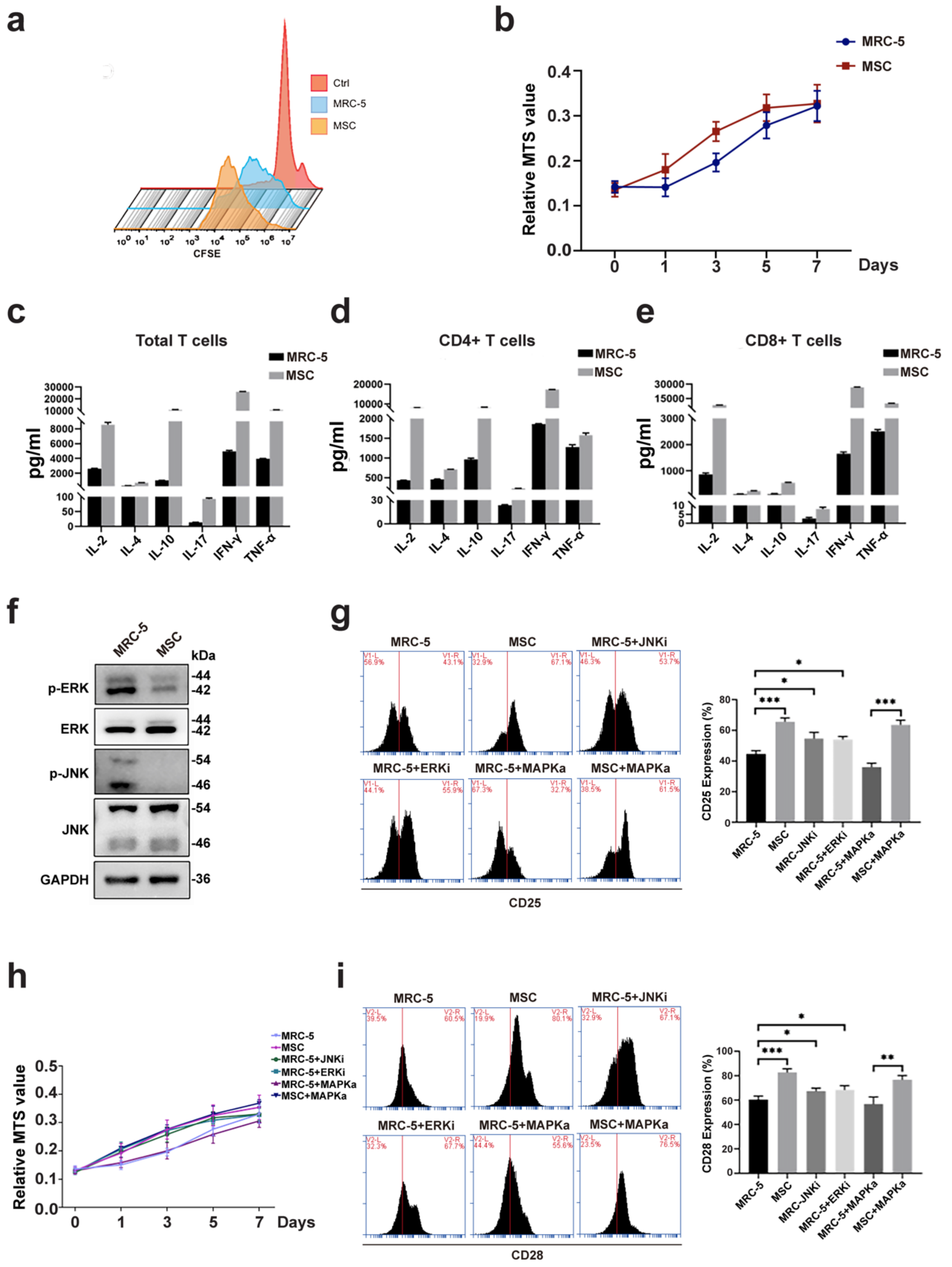

a
Ctrl
MRC-5
MSC
CFSE
b
MRC-5
MSC
Relative MTS value
Days
c
Total T cells
d
CD4+ T cells
e
CD8+ T cells
pg/ml
IL-2
IL-4
IL-10
IL-17
IFN-γ
TNF-α
f
p-ERK
ERK
p-JNK
JNK
GAPDH
kDa
g
MRC-5+JNKi
MRC-5+ERKi
MRC-5+MAPKa
MSC+MAPKa
CD25
CD25 Expression (%)
h
i
CD28
CD28 Expression (%)

Fig. S5 MSCs Support Immune Function and Promote CD28 Expression. a Overlay of representative flow cytometry data for the T-cell proliferation marker CFSE. **b** Line chart of MTS assay data for T-cell proliferation on seven consecutive days. **c–e** Cytokine secretion levels in the total T cells **c**, $CD4^+$ T cells **d**, and $CD8^+$ T cells **e**. **f** Western blotting of different proteins in T cells co-cultured with MRC-5 or MSCs. **g** Representative flow cytometry results (left) and summary histogram (right) of CD25 expression in activated T cells co-cultured with MRC-5/MSCs with or without different signaling inhibitors or activators. **h** Line chart of the MTS assay data for T cells co-cultured with MRC-5/MSCs with or without different signaling inhibitors or activators on seven consecutive days. **i** Representative flow cytometry results (left) and summary histogram (right) of CD28 expression in quiescent T cells co-cultured with MRC-5/MSCs with or without different signaling inhibitors or activators. The data represent the mean ± SD. The *P* values were determined using the unpaired Student t-test. *$P < 0.05$, **$P < 0.01$, ***$P < 0.001$. Related to Fig. 4.
