# Supplementary material for: Mesenchymal stem cell treatment improves outcome of COVID-19 patients via multiple immunomodulatory mechanisms
Source: Cell Res. 2021 Oct 26;31(12):1244–62. doi: 10.1038/s41422-021-00573-y (PMC8546390; doi:10.1038/s41422-021-00573-y)
Supplement: Supplementary file 6 — Supplementary Figure S6 [file 41422_2021_573_MOESM6_ESM.pdf]

Fig. S6

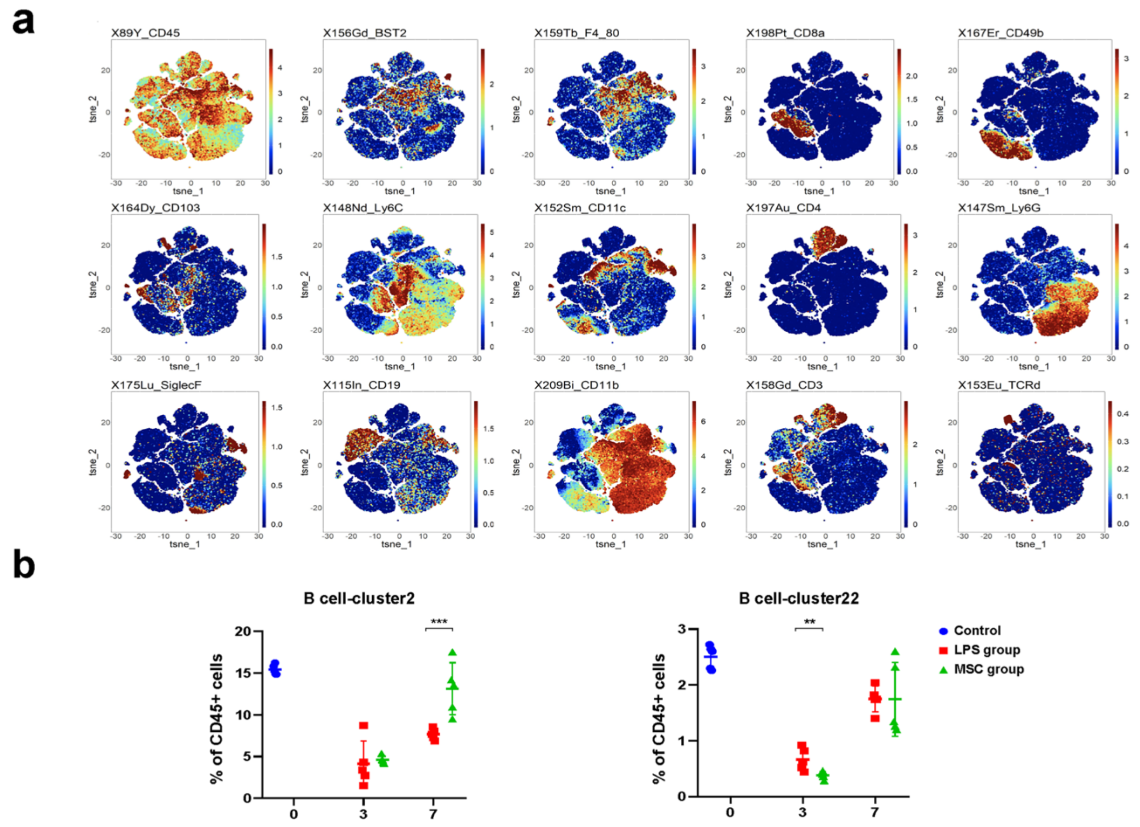

Fig. S6 Immune-Cell Markers and Proportions of B-Cell Subsets in LPS-treated and MSC-LPS-treated Mouse Lung. a viSNE map indicating the relative expression of each marker. **b** Percentages of B-cell subsets over time in LPS-treated and MSC-LPS-treated mouse lung. The data represent the mean ± SD. The p-values were determined using the unpaired Student t-test. $^{**}P < 0.01$, $^{***}P < 0.001$. Related to Fig. 5.
