# Supplementary material for: Mesenchymal stem cell treatment improves outcome of COVID-19 patients via multiple immunomodulatory mechanisms
Source: Cell Res. 2021 Oct 26;31(12):1244–62. doi: 10.1038/s41422-021-00573-y (PMC8546390; doi:10.1038/s41422-021-00573-y)
Supplement: Supplementary file 7 — Supplementary Table S1 [file 41422_2021_573_MOESM7_ESM.pdf]

Supplementary Table S1. Characteristics of healthy volunteers in the MSC infusion clinical trial.

Volunteer Number	Gender	Age
1	male	49
2	male	56
3	male	45
4	female	39
