# Supplementary material for: Mesenchymal stem cell treatment improves outcome of COVID-19 patients via multiple immunomodulatory mechanisms
Source: Cell Res. 2021 Oct 26;31(12):1244–62. doi: 10.1038/s41422-021-00573-y (PMC8546390; doi:10.1038/s41422-021-00573-y)
Supplement: Supplementary file 8 — Supplementary Table S2 [file 41422_2021_573_MOESM8_ESM.pdf]

Supplementary Table S2. Primers used in this study.

Gene	Primer	Sequence
IL-2	forward primer:	5’-AACTCCTGTCTTGCATTGCAC-3’
	reverse primer:	5’-GCTCCAGTTGTAGCTGTGTTT-3
IL-4	forward primer:	5’-CGGCAACTTTGTCCACGGA-3’
	reverse primer:	5’-TCTGTTACGGTCAACTCGGTG-3’
IL-10	forward primer:	5’-GAGGACTTTAAGGGTTACCTGGG-3’
	reverse primer:	5’-TCACATGCGCCTTGATGTCT-3’
IL-17	forward primer:	5’-TCCCACGAAATCCAGGATGC-3’
	reverse primer:	5’-TCACATGCGCCTTGATGTCT-3’
IFN-γ	forward primer:	5’-TCGGTAACTGACTTGAATGTCCA-3’
	reverse primer:	5’-TCACATGCGCCTTGATGTCT-3’
TNF-α	forward primer:	5’-GAGGCCAAGCCCTGGTATG-3’
	reverse primer:	5’-TCACATGCGCCTTGATGTCT-3’
GAPDH	forward primer:	5’-GGTCACCAGGGCTGCTTTTA-3’
	reverse primer:	5’-TCACATGCGCCTTGATGTCT-3'
SARS-CoV-2	forward primer:	5′-TCAGAATGCCAATCTCCCCAAC-3′
	reverse primer:	5′-AAAGGTCCACCCGATACATTGA-3′
	probe	5′CY5CTAGTTACACTAGCCATCCTTACTGC-3′BHQ1.
