# Supplementary material for: Mesenchymal stem cell treatment improves outcome of COVID-19 patients via multiple immunomodulatory mechanisms
Source: Cell Res. 2021 Oct 26;31(12):1244–62. doi: 10.1038/s41422-021-00573-y (PMC8546390; doi:10.1038/s41422-021-00573-y)
Supplement: Supplementary file 9 — Supplementary Table S3 [file 41422_2021_573_MOESM9_ESM.pdf]

Supplementary Table S3. Improvement of different clinical subtypes in the MSC-treated group and placebo-treated group.

Items		MSC group	Placebo group	*P* value[1]
**Clinical improvement rates**				
**Patients with common/mild COVID-19**				
**Day 7**				0.0909
	**Symptom remission***	7(24.1)	3(10.3)	
	**Improvement**	8(27.6)	10(34.5)	
	**No improvement**	0	3(10.3)	
**Day 14**				0.438
	**Symptom remission**	12(41.4)	10(34.5)	
	**Improvement**	3(10.3)	5(17.2)	
	**No improvement**	0	1(3.4)	
**Day 21**				0.2108
	**Symptom remission**	13(44.8)	15(51.7)	
	**Improvement**	2(6.9)	0	
	**No improvement**	0	1(3.4)	
**Patients with severe/critical COVID-19**				
**Day 7**				0.2507
	**Symptom remission**	4(13.8)	1(3.4)	
	**Improvement**	9(31.0)	9(31.0)	
	**No improvement**	1(3.4)	3(10.3)	
**Day 14**				0.0405
	**Symptom remission**	7(24.1)	2(6.9)	
	**Improvement**	6(20.7)	5(17.2)	
	**No improvement**	1(3.4)	6(20.7)	
**Day 21**				0.0157
	**Symptom remission**	8(27.6)	4(13.8)	
	**Improvement**	6(20.7)	3(10.3)	
	**No improvement**	0	6(20.7)	

1, The *P* value is obtained by the $\chi 2$ test.

*, This assessment includes patients whose symptoms have disappeared and patients discharged from hospital.

Data are n (%).
