# Supplementary material for: Mesenchymal stem cell treatment improves outcome of COVID-19 patients via multiple immunomodulatory mechanisms
Source: Cell Res. 2021 Oct 26;31(12):1244–62. doi: 10.1038/s41422-021-00573-y (PMC8546390; doi:10.1038/s41422-021-00573-y)
Supplement: Supplementary file 10 — Supplementary Table S4 [file 41422_2021_573_MOESM10_ESM.pdf]

Supplementary Table S4. Relationships between clusters and lung immune-cell populations.

Cell categorie	Cluster	Cluster No.
**T cell**	$CD4^+$ T cell	25
	$CD8^+$ T cell	5,21
	γδ T cell	19
**DC**	pDC	3
	$CD103^+$ DC	15
	DC	10
**Neutrophil**	-	1,4,6,9,17
**B cell**	IgM^+IgD^- B cell	22
	IgM^+IgD^+ B cell	2
**Eosinophil**	-	12,16
**Alveolar macrophage**	-	24
**Monocyte/macrophage**	-	7,8,11,18
**NK cell**	-	20,23
**Unknown**	-	13,14
