# Supplementary material for: Mesenchymal stem cell treatment improves outcome of COVID-19 patients via multiple immunomodulatory mechanisms
Source: Cell Res. 2021 Oct 26;31(12):1244–62. doi: 10.1038/s41422-021-00573-y (PMC8546390; doi:10.1038/s41422-021-00573-y)
Supplement: Supplementary file 11 — Supplementary Materials and Methods [file 41422_2021_573_MOESM11_ESM.pdf]

SUPPLEMENTARY METHODS

KEY RESOURCES TABLE

REAGENT or RESOURCE	SOURCE	IDENTIFIER
**Biological Samples**		
PBMCs from COVID-19 patients	YouAn hospital and Puren hospital	
Plasma from COVID-19 patients	YouAn hospital and Puren hospital	
Umbilical-cord MSCs	Qingdao Co-orient Watson Biotechnology Group, Co., Ltd.<br>Institute of Basic Medical Sciences, Chinese Academy of Medical Sciences	
scRNA-seq data of infusion MSCs		http://www.ncmi.cn/user_data.html
**Software and Algorithms**		
Cell Ranger (3.1.0)	10x Genomics	https://support.10xgenomics.com/single-cell-gene-expression/software/pipelines/latest/what-is-cell-ranger
Seurat (3.1.3)	Satija et al., 2015	https://satijalab.org/seurat/
SCSA	Cao et at., 2020	https://github.com/bioinfo-ibms-pumc/SCSA.
clusterProfiler (3.14.3)	Yu et al., 2012	https://guangchuangyu.github.io/software/clusterProfiler/
pysam (0.15.0)	Li et al., 2009	https://pysam.readthedocs.io/en/latest/
GATK (3.7)	The Broad Institute	https://github.com/broadgsa/gatk/releases/tag/3.7
PLINK (1.9)	Purcell et al., 2007	http://zzz.bwh.harvard.edu/plink/contact.shtml#cite
Flashpca (2.0)	Abraham et al., 2017	https://github.com/gabraham/flashpca
**Chemicals and Antibodies**		
PBS	Gibco	10010023
BSA	Thermo Scientific	23208
Chloral hydrate	Sigma-Aldrich	C8383-100G
LPS	eBioscience	00-4976-03
DMEM	Gibco	11965092
FBS	Gibco	16140071
IL-3	eBioscience	# 34-8031-82
IL-6	eBioscience	# 14-8061-80
SCF	eBioscience	# 14-8341-62

Transwell plates	Corning	CLS3412-24EA
RPMI 1640	Gibco	A1049101
Glutamine	Sigma-Aldrich	G7513
β-mercaptoethanol	Sigma-Aldrich	M3148
Non-essential amino acids	Sigma-Aldrich	TMS-001-C
IL-2	eBioscience	# RP-8605
phytohemagglutinin，PHA	Sigma-Aldrich	L1668
CD3	eBioscience	# 78-0032-80
CD69	eBioscience	# 14-0691-81
CD25	eBioscience	# 17-0251-81
CFSE living cells dye	BD Horizon	565082
MTS reagent	Promega	G1112
Human cytokine/chemokine magnetic bead panel kit	Millipore	HCYTOMAG-60K
TRIzol reagent	Invitrogen	15596018
SYBR premix Ex Taq	Takara	RR420A
RIPA lysis buffer	Thermo Scientific	89901
PMSF proteinase inhibitors cocktail	Beyotime	ST506
Phosphatase inhibitors cocktail	Yeasen	20104ES03
HRP-conjugated secondary antibodies	Neobioscience	16738
Chemiluminescent ECL reagent	Invitrogen	WP20005
Sytox Green solution	Invitrogen	S7020
BCA Protein Assay kit	Beyotime	P0012S
Reverse Transcription kit	Thermo Scientific	K1691
Agilent 2100 High Sensitivity DNA Assay kit	Agilent Technologies	5990-4417CHCN
Mouse SCA1 Positive Selection kit	Stemcell	18756RF
Cell lines		
MRC-5	Cell Bank of IBMS	N/A
